# Guide-based interventions aimed at reducing physical restraints in intensive care unit: a systematic review and meta-analysis of randomized controlled trials

**DOI:** 10.3389/fmed.2025.1606359

**Published:** 2025-09-26

**Authors:** Yueli Ping, Jianyan Yang, Yanming Zheng, Wanting Feng, Zexi Huang, Ruiqin Sha, Nianqi Cui, Ying Tian

**Affiliations:** ^1^School of Nursing, Kunming Medical University, Kunming, China; ^2^The First Affiliated Hospital of Kunming Medical University, Kunming, China; ^3^Department of Diabetes, The First Affiliated Hospital of Kunming Medical University, Kunming, China; ^4^Department of Nursing, The First Affiliated Hospital of Kunming Medical University, Kunming, China

**Keywords:** restraint, physical, intensive care units, critical care nursing, guidelines as topic, evidence-based practice

## Abstract

**Objective:**

Despite widespread advocacy and organizational support for reducing the utilization of physical restraint (PR) in clinical settings, its application remains prevalent on a global scale. This study aims to identify and evaluate guide-based, high-quality interventions that can be effectively integrated into clinical practice to substantially reduce PR utilization rates.

**Methods:**

A comprehensive search of relevant databases was covered all available records from their establishment through November 10, 2024, including PubMed, the Cochrane library, Web of Science, CINAHL, EMBASE, the Joanna Briggs Institute (JBI), China National Knowledge Infrastructure (CNKI), Wanfang Data, China Science and Technology Journal Database (VIP), and Chinese BioMedical Literature Service System (SinoMed). The search specifically targeted randomized controlled trials (RCTs) that focused on guide-based interventions designed to reduce the utilization of PR in the intensive care unit (ICU). Two independent researchers systematically reviewed the literature, with each investigator independently extracting relevant data and assessing the methodological quality of included studies using standardized criteria. The subsequent meta-analysis was conducted using Review Manager software version 5.2.

**Results:**

A total of 14 RCTs, involving 4,338 participants, were included in the analysis. The results indicated that guide-based interventions significantly reduced the PR rate (RR = 0.72, *P* < 0.001), PR time [weighted mean differences (WMD) = −248.5, *P* = 0.002], delirium incidence (RR = 0.53, *P* < 0.001), duration of delirium (WMD = −11.94, *P* = 0.008), unplanned extubation rate (RR = 0.36, *P* < 0.001), the other complications rate (RR = 0.36, *P* < 0.001), and duration of mechanical ventilation (WMD = −31.84, *P* = 0.005). Notably, in contrast to other outcomes, these interventions were associated with increased patient satisfaction (RR = 1.16, *P* < 0.001). However, there was no evidence to suggest that guide-based interventions reduced the length of ICU stay or patient agitated or anxiety rate (*P* > 0.05).

**Conclusion:**

Guide-based interventions can effectively reduce the utilization of PR with patients in ICU. Employing a multidisciplinary team, adjusting patient assessment frequency by PR type and standardizing the PR assessment scale are possible to reduce the utilization of PR.

**Systematic review registration:**

https://www.crd.york.ac.uk/PROSPERO/view/CRD42024623625, identifier: CRD42024623625.

## 1 Introduction

Physical restraint (PR) is defined as “Any intervention or procedure that intentionally restricts an individual's free body movement through the application of any method, device, or apparatus that cannot be easily removed or controlled by the individual” (1). The utilization of PR in intensive care units (ICUs) demonstrates significant global prevalence, a trend that is consistently documented across diverse healthcare settings worldwide. In Japan, 85.6% of 787 patients in six ICUs underwent PR (2). In China, 61.2% of 312 patients in three ICUs underwent PR (3). In Canada, 52.6% of 711 patients across 51 ICUs in 10 provinces underwent PR (4). The high utilization of PR among ICU patients is attributable to the comprehensive nature of medical systems designed to manage critically ill individuals, which often necessitate invasive procedures such as catheter placement and mechanical ventilation (5, 6). Nurses typically employ PR as a preventative measure to avert patient harm, specifically to prevent unplanned extubation in ICUs (7). Nonetheless, a two-center study found that anxious, agitated patients may try to remove uncomfortable tubes, resulting in unplanned extubation (8). Meanwhile, ongoing research shows a strong link between PR use and both physical and psychological issues in patients. PR has been clinically associated with various neurovascular complications (e.g., localized erythema, restricted limb mobility, peripheral edema, and alterations in skin coloration) (9), pressure injuries (10), delirium (11) and increased length of stay (12). A qualitative systematic review found that patients undergoing PR often experience significant psychological distress, including anger, fear, physical discomfort, and a sense of lost dignity, along with feelings of dehumanization and reduced self-worth (13). Since 2003, numerous organizations—including American College of Critical Care Medicine, the British Association of Critical Care Nurses and Chinese Nursing Association—have advocated and supported reducing the utilization of PR in clinical practice (14–16).

In the Oxford Dictionary, guide is defined as “Something that helps you to make a judgment about something.” In the field of healthcare and nursing, guide is typically seen as tools or methods that assist individuals or groups in making decisions under specific circumstances (17). These guides can take various forms, such as Clinical Practice Guidelines (CPGs), nursing bundle and syntheses of best evidence. CPGs are a common form of these, representing a standardized form of evidence-based recommendations, comprising systematically developed statements designed to optimize patient care outcomes. These are developed by thoroughly evaluating clinical evidence, including systematic reviews and risk-benefit analyses of alternative treatments (18). The using guide-based interventions are possibly effective when developing interventions to reduce PR. A randomized controlled trial (RCT) showed that patients who received PR interventions that are based on syntheses of best evidence had significantly lower PR rate, PR time, and incidence and duration of delirium, etc (19). Another study indicated that patients who received PR interventions that are based on PR decision wheel had lower PR rate, but the rates of unplanned extubation and other complications remained unchanged (20). Guide-based interventions could offer a way to decrease PR use, reduce harm, and improve patient safety. However, despite promising trends, it's still necessary to systematically assess if PR in ICUs can truly be reduced.

In summary, the objective of this study is to systematically review and critically appraise guide-based interventions aimed at reducing PR by analyzing RCTs and to identify high-quality interventions that can be implemented in clinical practice to effectively reduce the PR rate.

## 2 Methods

The meta-analysis was conducted in strict accordance with the Preferred Reporting Items for Systematic Reviews and Meta-Analyses (PRISMA) guidelines (21). This study was conducted as a retrospective analysis exclusively utilizing published research data, thereby eliminating the need for direct human subject involvement. In accordance with established institutional protocols and ethical guidelines, formal review by the Institutional Review Board was deemed unnecessary. In order to guarantee transparency and maintain a high level of methodological rigor, the study protocol was registered in advance in the PROSPERO international prospective register of systematic reviews before the research was initiated (CRD42024623625).

### 2.1 Search strategy

A comprehensive search of relevant databases was covered all available records from their establishment through November 10, 2024. The databases researchers searched were: PubMed, the Cochrane library, Web of Science, CINAHL, EMBASE, the Joanna Briggs Institute (JBI), China National Knowledge Infrastructure (CNKI), Wanfang Data, China Science and Technology Journal Database (VIP) and Chinese BioMedical Literature Service System (SinoMed) and a manual search was carried out for relevant literature sources (For more in-depth details, please refer to Supplementary Appendix A). To maximize study identification and ensure methodological rigor, we implemented a multi-faceted approach that included: systematic examination of previously published reviews, meticulous scrutiny of reference lists from all included studies, and critical analysis of existing meta-analyses to identify potentially eligible articles that might have been overlooked through conventional search methods.

### 2.2 Inclusion and exclusion criteria

The PICOS framework, which encompasses population, intervention, comparison, outcome, and study design, was employed to formulate stringent inclusion criteria for study selection (22). The inclusion criteria were established as follows: (1) population. ICUs patients (≥18 years old); (2) intervention. Experimental group received guide-based interventions; (3) comparison. Control group implemented nursing procedures as usual; (4) outcomes. Primary outcome is PR rate or PR time. Secondary outcome is to evaluate unplanned extubation rate, delirium incidence and other complications rate; (5) study design. RCT, published in peer-reviewed journals, with language restrictions limited to English and Chinese publications.

Exclusion criteria were established as follows: (1) studies in which the intervention was poorly described, making it impossible to determine if it was a “guide-based intervention.”; (2) outcome measures were incomplete and data could not be extracted for meta-analysis; (3) reviews, case reports, cohort studies, cross-sectional studies, etc; (4) abstract-only articles; (5) literature that is duplicated, incomplete or incorrect.

### 2.3 Date extraction

The citations of all the studies obtained from the search were imported into the reference management software, Endnote X9. Subsequently, two researchers, (the primary and the co-primary authors) independently evaluated the methodological quality and relevance of the retrieved studies. This evaluation was carried out in strict accordance with the pre-established inclusion and exclusion criteria. Data extracted from each study included the authorship, year of publication, country of origin, sample size, details of the guide-based intervention, study settings, outcome measures, and principal findings. Any discrepancies identified between the two researchers during the evaluation process were systematically addressed through iterative discussion, with unresolved disagreements being referred to a third senior researcher for final arbitration.

### 2.4 Quality assessment of included studies

Two independent researchers (the primary and co-primary authors) conducted a rigorous assessment of bias risk and methodological quality in the included RCTs, following the standardized criteria established in the Cochrane Handbook for Systematic Reviews of Interventions (23). The evaluation encompassed seven critical domains of potential bias: randomization sequence generation, allocation concealment, participant blinding, outcome assessment blinding, insufficient outcome data, selective reporting, and other sources of bias.

### 2.5 Statistical analysis

The meta-analysis was conducted using Review Manager (RevMan) software, version 5.2. When dealing with dichotomous outcome measures, the effect magnitudes were represented as risk ratios (RR) along with the corresponding 95% confidence intervals (CI). In contrast, for continuous outcome variables, the analysis made use of weighted mean differences (WMD) together with their 95% CI. Statistical heterogeneity was assessed through multiple indicators, including chi-square test, *I*^2^, and *P* value. A fixed-effects model was applied when heterogeneity was deemed acceptable (*P* > 0.1 and *I*^2^ < 50%), whereas a random-effects model was implemented in cases of significant heterogeneity (*P* < 0.1 or *I*^2^ ≥ 50%), except when studies demonstrated substantial clinical homogeneity. To evaluate the robustness of findings and identify potential sources of heterogeneity, sensitivity analyses were performed through sequential exclusion of individual studies. The assessment of publication bias was carried out by analyzing the symmetry of the funnel plot. Additionally, when the analysis involved more than 10 studies, formal statistical tests, namely Begg's and Egger's tests, were conducted using Stata statistical software (version 18.0; StataCorp LP, College Station, TX) to supplement the funnel plot analysis.

## 3 Results

### 3.1 Description of included studies

**Figure 1:**
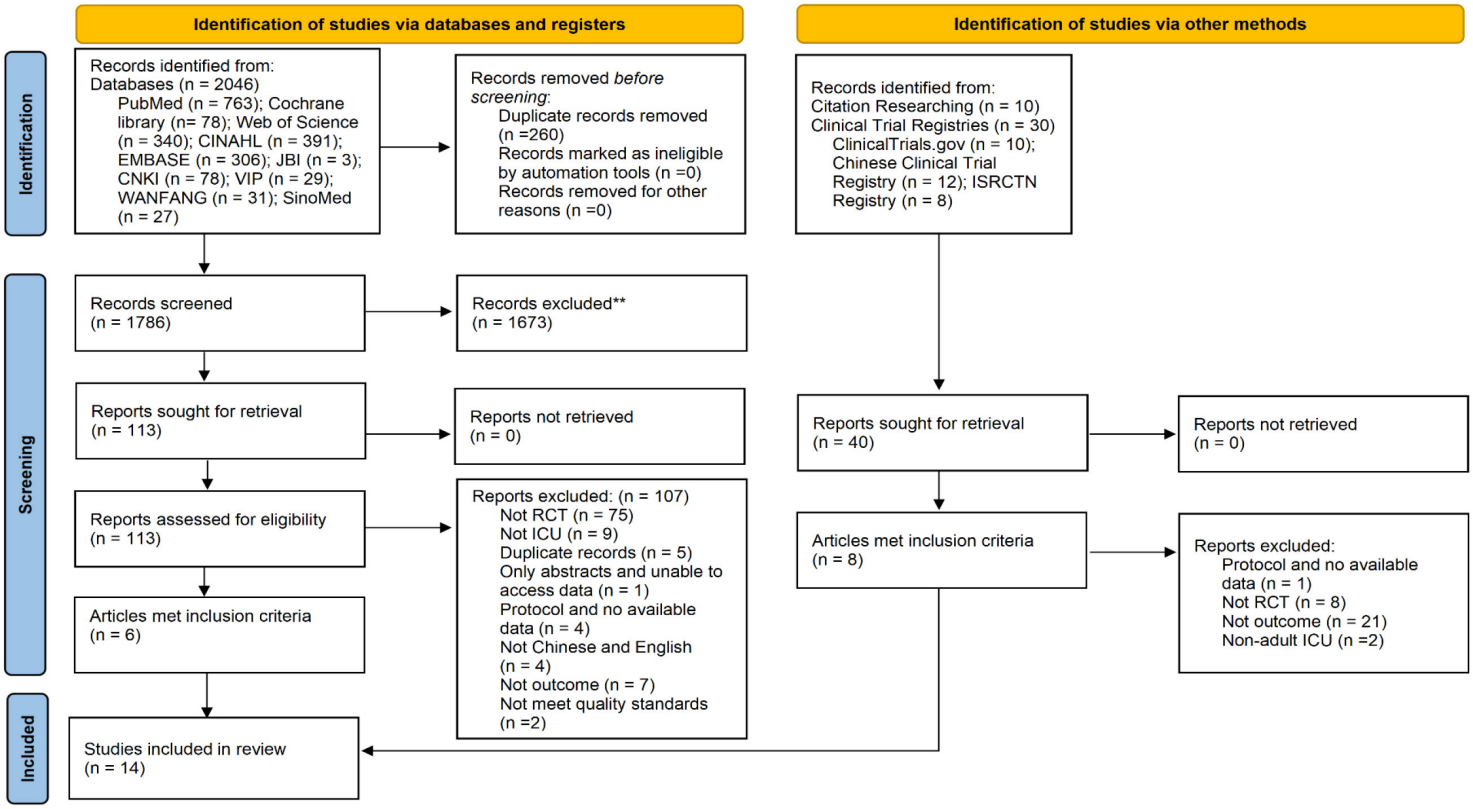
Flowchart of study selection process.

The systematic review ultimately included 14 studies (19, 20, 24–35), of which 13 were conducted in China and one in Colombia. Figure 1 presents a detailed flowchart of the search and selection process. The included studies mainly investigated adult patients in ICU, with patient recruitment and intervention implementation occurring across various ICU subtypes, including integrated ICUs, general ICUs, and neurological ICUs. Comprehensive characteristics of the included studies, along with their primary outcomes, are systematically presented in Table 1.

**Table 1:** Characteristic and outcomes of included studies.

**Author, year, and country**	**Patient characteristics**	**Sample size (EG/CG)**	**Settings**	**Outcome measures**	**Main results**
Song et al. (20) (2015) China	ICU adult consciousness disorder patients	129/125	ICU of Dongguan Eighth People's Hospital in July 2012 to December 2013		There was only significant difference of PR rate (73.60 vs. 48.84%, *P* < 0.01)
Wu et al. (30) (2019) China	ICU adult catheterized patients	250/182	Integrated ICU, Respiratory ICU, Cardiovascular Medicine ICU, Cardiothoracic Surgery ICU, Neurology ICU, Neurosurgery ICU of The Affiliated Hospital of Nantong University in July 2018 to August 2018	①⑥⑦	With the interventions being applied, the PR rate in the EG had lower (90.00 vs. 74.00%, *P* < 0.001) and shorter PR time (643.53 ± 388.30 vs. 529.25 ± 417.00, *P* = 0.004)
Yan et al. (32) (2019) China	ICU adult catheterized patients	55/55	ICU of Tongzhou, Nantong City, Jiangsu Province District Hospital in June 2017 to June 2018	①②③④⑤⑥⑦⑧⑨⑩⑪␑␒␓	The PR time of EG shorter than CG (45.02 ± 4.56 vs. 33.25 ± 3.02, *P* < 0.001) and there was significant difference of unplanned extubation rate (18.18 vs. 3.63%, *P* = 0.014)
Chen (26) (2019) China	ICU adult consciousness disorder patients	30/30	ICU of the First Affiliated Hospital of Henan University of Science and Technology in May 2016 to May 2018	②⑥⑦	There existed a substantial variation in the PR rate (96.67 vs. 70.00%, *P* = 0.006) and unplanned extubation rate (30.00 vs. 3.33%, *P* = 0.006)
Yu et al. (34) (2019) China	ICU adult mechanical ventilation patients	35/33	ICU of the Second People's Hospital of Wuxi in December 2016 to December 2017	①⑥⑦	There existed a substantial variation in the PR rate (53.28 vs. 43.64%, *P* = 0.009) and delirium incidence (51.52 vs. 25.71%, *P* = 0.029)
Qian et al. (27) (2020) China	ICU adult consciousness disorder patients	60/60	ICU of People's Hospital of Hai'an City, Jiangsu Province in February 2018 to February 2019	①⑤␘␙␠	There existed a substantial variation in the PR rate (95.00 vs. 70.00%, *P* < 0.05) and delirium incidence (51.52 vs. 25.71%, *P* < 0.05)
Wu et al. (24) (2021) China	ICU adult mechanical ventilation patients	133/133	Integrated ICU of The Affiliated Hospital of Zunyi Medical University in January 1 to December 31, 2020	①⑥⑦	There existed a substantial variation in the PR rate (45.10 vs. 19.50%, *P* < 0.001) and the PR time of EG shorter than CG (13.55 ± 7.40 vs. 9.71 ± 4.07, *P* < 0.001)
Zhang et al. (35) (2021) China	ICU adult catheterized patients	120/120	Integrated ICU of The First Affiliated Hospital of Zhengzhou University in February 2018 to January 2019	①②⑤␉	There existed a substantial variation in the PR rate (70.83 vs. 46.66%, *P* = 0.001) and the PR time of EG shorter than CG (41.24 ± 11.36 vs. 30.42 ± 12.52, *P* < 0.001)
Yang (33) (2021) China	ICU adult catheterized patients	43/43	ICU of The First Affiliated Hospital of Henan University of Science and Technology in October 2018 to October 2020	①②③④⑤⑥⑦⑧⑨⑩	There existed a substantial variation in the PR rate (76.74 vs. 46.51%, *P* = 0.004) and unplanned extubation rate (18.60 vs. 4.65%, *P* = 0.044)
Xu et al. (31) (2022) China	ICU adult consciousness disorder patients	97/96	ICU in March 2019 to July 2020	①②③④⑤⑥⑦⑧⑨⑩	There existed a substantial variation in the PR rate (65.63 vs. 48.45%, *P* = 0.014) and the PR time of EG shorter than CG (57.36 ± 7.15 vs. 51.43 ± 7.20, *P* < 0.001)
Wang et al. (29) (2022) China	ICU adult mechanical ventilation patients	57/56	ICU of A tertiary hospital in Huangshan City, Anhui Province in February 2021 to February 2022	①②③④⑤⑥⑦⑧⑨⑩	There was no significant difference of PR rate and unplanned extubation rate; the PR time of EG shorter than CG
Tao et al. (28) (2023) China	ICU adult mechanical ventilation patients	96/96	ICU of Shanghai Sixth People's Hospital in March 2020 to February 2022	①②	There existed a substantial variation in the PR rate (76.09 vs. 47.83%, *P* = 0.005) and delirium incidence (23.91 vs. 8.70%, *P* = 0.048)
Yang et al. (25) (2023) China	ICU adult catheterized patients	682/1309	General ICU, Neurosurgery ICU, Neurology ICU and Cardiac Surgery ICU of Affiliated Hospital of Nantong University in		Compared with pre-implementation and post-implementation, the PR time was shortened (682.16 ± 370.81 vs. 467.41 ± 406.37; *P* = 0.000) and PR rate was decreased (91.2 vs. 73.7%; *P* = 0.000)
Gómez Tovar et al. (19) (2024) Colombia	ICU adult	71/142	ICUs in a university hospital in Colombia in August 2021 and February 2022	③⑤␔␙␡	Comparing groups of study, the delirium incidence was lower in the EG than in the CG (14.8 vs. 5.6%, *P* = 0.037) and lower PR time (1.27 ± 0.46 vs. 1.21 ± 0.24, *P* = 0.06)

EG, experimental group; CG, control group;① PR rate;② PR time;③ analgesic rate;④ sedation rate;⑤ delirium incidence;⑥ unplanned extubation rate;⑦ the other complications rate (redness, oedema, color complication, pressure injure and the limb not in a functional position);⑧ patient anxiety rate;⑨ negative psychological reaction between the family members;⑩ PR-related or unplanned extubation knowledge of doctors and nurses; ⑪ the standard rate of utilization of PR devices; ⑫ lighting management execution rate; ⑬ the pass rate of environmental cleanliness; ⑭ noise, temperature, humidity pass rate. ⑮ treatment cooperation rate; ⑯ patient agitated rate; ⑰ comfort score; ⑱ patient satisfaction; ⑲ length of stay in the ICU; ⑳ duration of mechanical ventilation; ⑴ dosage of sedation; ⑵ duration of delirium; ⑶ patient depression disorder; ⑷ the sleep quality; ⑸ dosage of analgesic; ⑹ mortality.

### 3.2 Details of interventions of included studies

These guides of included studies took various forms, such as CPGs, ABCDEF bundle and syntheses of best evidence. The measurement instruments utilized during the intervention predominantly originate from guidelines available on official websites or are authored by experts, supplemented by some self-compiled assessment scales. Implementation was carried out by multidisciplinary healthcare teams comprising physicians, critical care specialist nurses, and clinical technicians. Intervention efficacy demonstrated significant variability based on program-specific configurations and their corresponding implementation parameters. Detailed characteristics of all guide-based interventions, including their specific components and implementation interventions, are presented in Table 2.

**Table 2:** Guide-based intervention details of included studies.

**Study**	**Guide**	**Team members**	**Frequency of assessments**	**Measuring materials**	**Main procedures**
Song et al. (20)	JCAHO (CPG), PR Decision Wheel and Assessment of PR (36)	• It is not mentioned in the article	• Comprehensive assessment (each/8 h); • Sedated patients (each/4 h) and agitated patients (each/15 min)	PR Decision Wheel (Behavior Level, Facility Level, Independence Level, PR Level)	• Based on the PR decision wheel to decide PR methods (If the evaluation results of behavior, facility, and independence in three aspects all should adopt PR, only then can the PR be implemented, otherwise, they can't adopt PR or should adopt alternative methods); • Comprehensive assessment before PR: determine the necessity of PR and report to the doctor; • Ongoing assessment.
Wu et al. (30)	Syntheses of best evidence	• EG: 82 critical care nurses and 19 intensivists • CG: 53 critical care nurses and 24 intensivists	• Check the vital signs, skin and blood supply of the PR area (each/1 h); • Re-evaluate the PR necessity (each/8 h); • Comprehensive assessment on shift handover	PR Decision Wheel; Assessment of PR; ICU inpatient PR assessment scale	• Build a PR flow diagram; • Make training manuals; • Shoot a video; • PR knowledge training; • Revised informed consent forms and health education manuals; • Select PR assessment tools; • Updated Doctor's order entries.
Yan et al. (32)	JCAHO (CPG), Assessment of PR (36)	PR assessment team: 1 head nurse of the department, 1 ward nurse manager, 1 critical care nurse and 2 nurses familiar with the PR process.	• Assess the tightness of the patient's PR (each/2 h); • Re-evaluated PR each shift using the PR-reduction protocol	Manual muscle testing (Lovett scale); RASS	• Establish a PR assessment team; • PR reduction protocol: • I. No PR; • II. Alternative PR; • III. Partial PR. • IV. Full PR.
Chen (26)	JCAHO (CPG), PR Decision Wheel and Assessment of PR (36)	It is not mentioned in the article	• PR patient: Ongoing assessment; • No PR: re-evaluate at shift handover; • Patients who may be released from PR: every 8 h evaluate 1 time; • Sedated patients (each/4 h) and agitated patients (each/15 min)	PR Decision Wheel	• Based on the PR decision wheel to decide PR methods; • Comprehensive assessment before PR: determine the necessity of PR and report to the doctor; • Ongoing assessment.
Yu et al. (34)	ABCDEF bundle	It is not mentioned in the article	• Dynamically assessed during daily morning rounds	RASS; CPOT	• Early activity: full range of joint motion, sitting exercise, bed-free activities, walking exercises; • Occupational therapy: ADLs, functional occupational therapy.
Qian et al. (27)	JCAHO (CPG), PR Decision Wheel and Assessment of PR (36)	It is not mentioned in the article	• Comprehensive assessment (each/8 h); • Sedated patients (each/4 h) and agitated patients (each/15 min)	PR Decision Wheel	• Based on the PR decision wheel to decide PR methods; • Comprehensive assessment before PR: determine the necessity of PR and report to the doctor; • Ongoing assessment
Wu et al. (24)	ABCDEF bundle	• The department director and head nurse: full responsibility for supervision and coordination; • Rehabilitation nurse: implementation; • Bedside doctor and charge nurse: assist	• Comprehensive assessment (each/8 h)	• RASS; CPOT; CAM-ICU; manual muscle testing (Lovett scale)	• Early bed-free activities: bedside wheelchair sitting or walker standing, walking, and the time of bed-free activities until the patient is intolerant
Zhang et al. (35)	JCAHO (CPG), PR Decision Wheel and Assessment of PR (36)	The department director, the head nurse, 3 critical care nurses, and 3 nurses with more than 3 years of experience in PR nursing work.	• Assess the tightness of the patient's PR (each/2 h); • Re-evaluated PR each shift using the PR-reduction protocol	Manual muscle testing (Lovett scale); RASS	• Establish a PR assessment team; • PR reduction protocol: • I. No PR; • II. Alternative PR; • III. Partial PR. • IV. Full PR.
Yang (33)	JCAHO (CPG), PR Decision Wheel and Assessment of PR (36)	Head nurse, 7 critical care nurses	• Assess the tightness of the patient's PR (each/2 h); • Re-evaluated PR each shift using the PR-reduction protocol	Manual muscle testing (Lovett scale)	• Establish a PR assessment team; • PR reduction protocol: • I. No PR; • II. Alternative PR; • III. Partial PR. • IV. Full PR.
Xu et al. (31)	• JCAHO (CPG), • PR Decision Wheel and Assessment of PR (36) • treatment interference protocol (37) • Chinese Nursing Association Inpatient PR care (16)	Two intensivists, one head nurse and 10 critical care nurses	• No PR: each/24 h; • Selective PR: each/8 h; • Full PR: each/2 h	Self-compiled ICU Patient PR Assessment Scale	• Establish a PR assessment team; • Score 0–24 (No PR— < 14, Selective PR −14–19, Full PR—>19); • Nurses' PR knowledge and skills training and examination; • Ongoing assessment
Wang et al. (29)	Branch Of Critical Care Medicine, Chinese Medical Association (CPG), Guideline for the management of pain and sedation in adult patients in the ICU (38)	More than 5 years of work in ICU, through RASS and CAM-ICU related training for critical care nurses	• Comprehensive assessment (each/8 h); • Assess the tightness of the patient's PR (each/2 h)	RASS, CAM-ICU	• Establish a PR assessment team; • RASS (No PR—−4–−5, Selective PR—−3–1, Full PR −2–4); • Ongoing assessment
Tao et al. (28)	ABCDEF bundle	1 intensivist, 1 respiratory therapist, 1 rehabilitation therapist, 1 psychologist and 6 critical care nurses. The head nurse of the department serves as the team leader	• Daily awaken test: 9:00 a.m. every day, respiratory therapist and responsible nurse; • Spontaneous breathing test: after daily awaken test is successful, respiratory therapist and attending physician; • Sedative and analgesic drug use: Shift handover for each class (3 times daily), attending physician and responsible nurse; • Delirium assessment and prevention: RASS ≤ -3, attending physician and responsible nurse share the use of CAM-ICU, if there is inconsistency, consult a psychiatrist; • Early activities: 3 times daily, responsible nurses and rehabilitation therapist; • Family engagement and empowerment: 4:00 p.m.	RASS, CAM-ICU, CPOT, RCSQ	• Establish a PR bundle team; • Training and examination; • ABCDEF bundle
Yang et al. (25)	Syntheses of best evidence	It is not mentioned in the article	• Check the vital signs, skin and blood supply of the PR area (each/1 h); • Re-evaluate the PR necessity (each/8 h); • Comprehensive assessment on shift handover	PR Decision Wheel; Assessment of PR; ICU inpatient PR assessment scale	• Build a PR flow diagram; • Make training manuals; • Shoot a video; • PR knowledge training; • Revised informed consent forms and health education manuals; • Select PR assessment tools; • Updated Doctor's order entries.
Gómez Tovar et al. (19)	• Scoping review: • I. nursing theory— “Dynamic Symptoms Model” • II. from the empirical approach with the scientific evidence of non-pharmacological care	It is not mentioned in the article	• Assess signs of pain(each/2 h); • Daily sedation goal (each shift), RASS evaluation (each/2 h); • Orientation on the date, time, and place (each shift); • CAM-ICU: every shift	RASS, CAM-ICU, VAS, Campbell scale (non-communicable patients)	• Family companionship and support; • Check for pain; • Early mobility and exercise; • Cognitive Stimulation; • Encourage preferences to reduce stress; • Identify and solve spiritual, social and environmental needs.

JCAHO, Joint Commission on Accreditation of Health care Organizations; RASS, The Richmond Agitation-Sedation Scale; I. No PR: RASS ≤ -4, Lovett ≤ 2 and conscious; II. Alternative PR: Lovett>2, confusion, tubing number ≥2 and basically cooperate with treatment; III. Partial PR: RASS ≤ -3 or 0, Lovett>2 and history of cerebrovascular disease; IV. Full PR: Lovett>2, the patient is very agitated and cannot cooperate with the treatment; ABCDEF bundle: A-Assess, prevent, and manage pain; B-Both spontaneous awakening trials and spontaneous breathing trials; C- Choice of analgesia and sedation; D- Delirium assessment, prevention and management; E- Early mobility and exercise; F- Family engagement and empowerment; CPOT, Critical Care Pain Observation Tool; Organization ADLs, Activity of Daily Living Scale, such as wash etc; CAM-ICU, Confusion Assessment Model for Intensive Care Unit; RCSQ, Richards-Campbell Sleep Questionnaire; VAS, Visual Analog Scale.

### 3.3 Details of interventions of included studies

**Figure 2:**
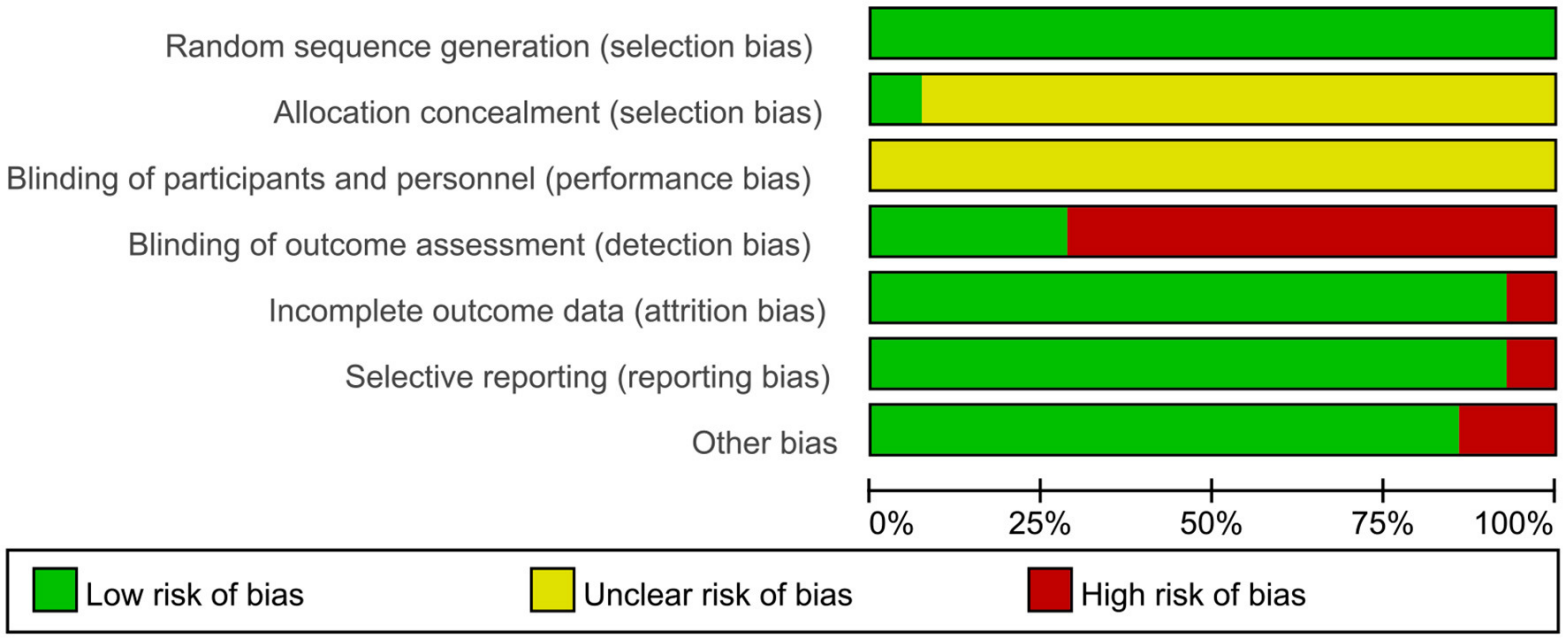
Graph the risk of bias. Evaluations for each category of bias risk are presented as percentages across all the studies that have been included.

**Figure 3:**
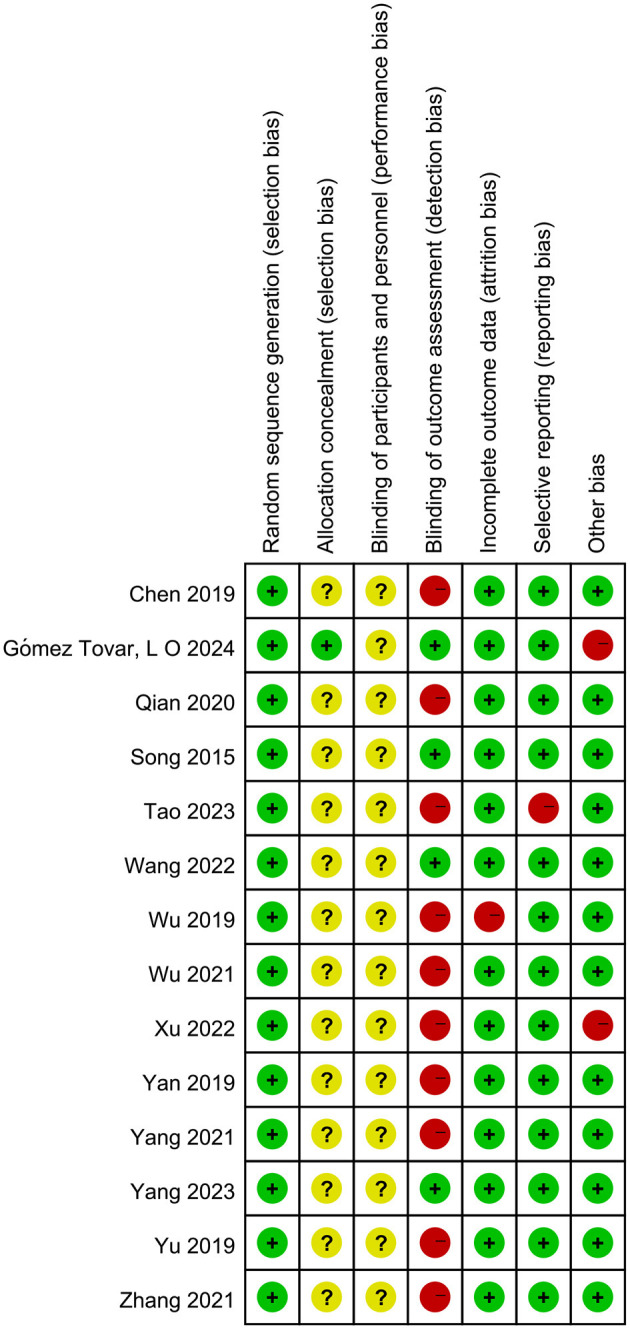
Summary of the risk of bias. Evaluations of every bias risk category conducted for individual studies. (“+” means low risk; “–” means high risk; “?” means unclear risk).

The methodological quality assessment of included studies, as presented in Figures 2, 3, revealed the following findings regarding risk of bias: among the 14 studies analyzed, random sequence generation methods varied significantly, with nine studies utilizing random number tables, two cluster RCTs employing drawing lots administered by ICU head nurses, one study using sealed envelopes, one study implementing a block randomization list, and one study applying stratified random sampling. Only a single study demonstrated appropriate allocation concealments. The majority of studies (*n* = 13) failed to implement blinding of participants and personnel, reflecting the practical challenges associated with masking guide-based interventions in clinical settings. Four studies reported adequate blinding procedures for outcome assessment. One study was classified as having high risk of bias due to incomplete outcome data. Regarding reporting bias, 13 studies showed low risk of selective outcome reporting, while 12 studies demonstrated low risk of other potential biases.

### 3.4 Meta-analysis results

#### 3.4.1 Effect of guide-based interventions on the PR rate and PR time

**Figure 4:**
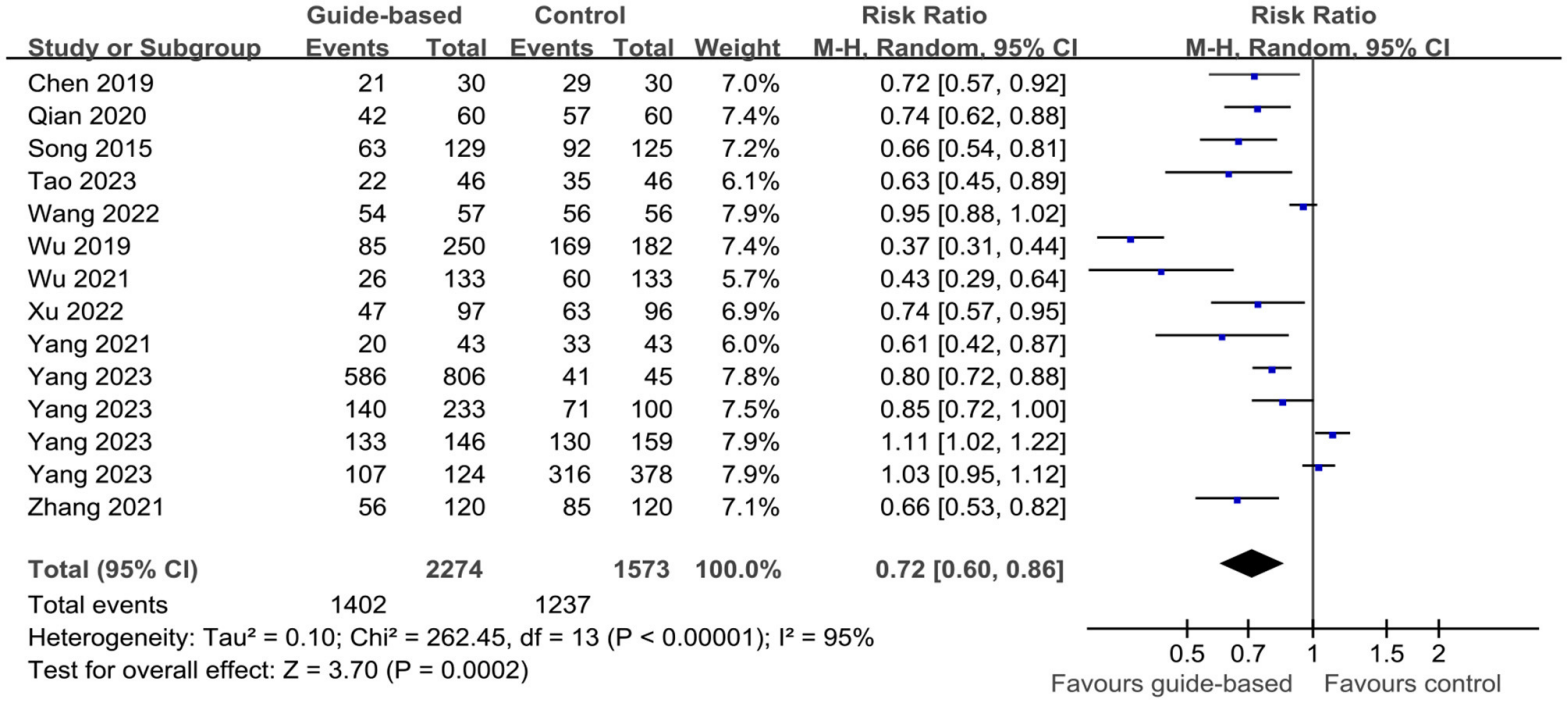
Effect of guide-based interventions on the PR rate with patients in ICU.

**Figure 5:**
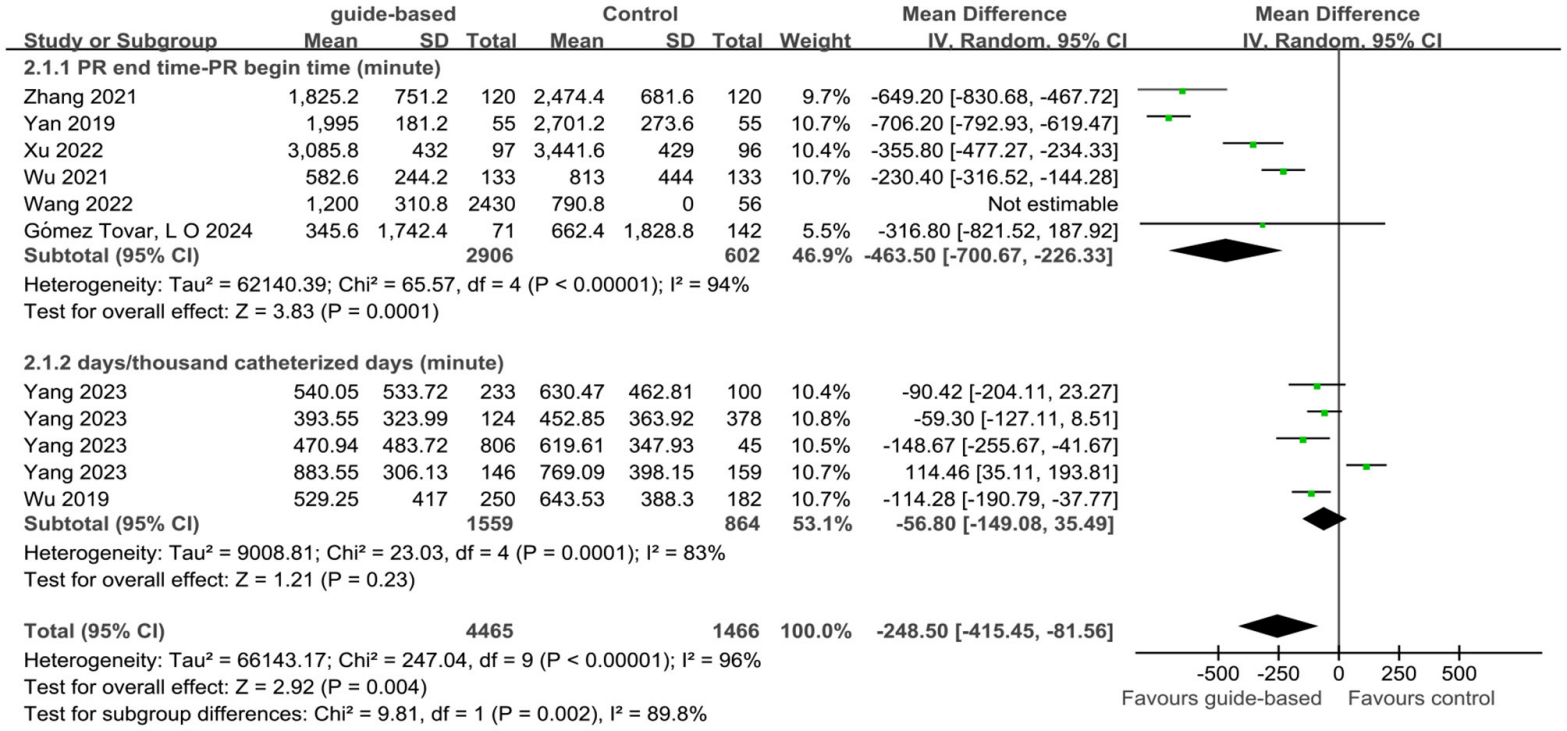
Effect of guide-based interventions on the PR time with patients in ICU.

Twelve studies (20, 24–31, 33–35) of 14 studies assessed the impact of guide-based interventions on the PR rate among ICU patients. For the PR rate, Yu et al.'s (34) calculation formula was (PR days/patient days × 100%), but the remaining 11 studies were calculated as (the number of physical restraints/total number of patients). Meta-analysis was performed on the remaining 11 studies due to inconsistencies in the calculation formulas. Using a random-effects model, the analysis revealed that the PR rate in the experimental group was 0.72 times than that in the control group (RR = 0.72, 95% CI 0.60 to 0.86, *P* < 0.001; *I*^2^ = 95%). Additionally, eight studies (19, 24, 25, 29–32, 35) of 14 studies assessed the impact of guide-based interventions on PR time among ICU patients. Due to substantial methodological heterogeneity, a subgroup analysis was pre-specified based on the formula used to calculate PR time. The rationale for this analysis was that the included studies employed two distinct metrics: (1) “PR end time minus PR begin time,” which measures a single PR time at the individual level, and (2) “PR days/thousand catheterized days,” which measures the frequency of restraint use at the unit level. Pooling these clinically and methodologically different outcomes was not appropriate; therefore, we analyzed their effects separately. Subgroup analyses were performed to account for variations in calculation formulas. The random-effects model demonstrated that guide-based interventions significantly reduced PR time compared to control groups (WMD = −248.5, 95% CI −415.45 to −81.56, *P* = 0.002, *I*^2^ = 89.8%; Table 3, Figures 4, 5).

**Table 3:** Effects of guide-based interventions on clinical outcomes with patients in ICU.

**Outcomes**	**Number of studies**	**Number of patients**	**Statistical method**	**Effect estimate**	***I*^2^ value (%)**	***P-*value**
PR rate	11	3,847	Mantel-Haenszel, random	0.72 [0.60, 0.86]	95	< 0.001
PR time	8	3,445	Inverse variance, random	−248.50 [−415.45, −81.56]	89.8	0.002
Delirium incidence	6	1,062	Mantel-Haenszel, fixed	0.53 [0.41, 0.68]	0	< 0.001
Duration of delirium	3	571	Inverse variance, random	−11.94 [−20.75, −3.13]	89	0.008
Unplanned extubation rate	10	1,700	Mantel-Haenszel, fixed	0.36 [0.23, 0.56]	0	< 0.001
Other complications rate	9	1,608	Mantel-Haenszel, fixed	0.36 [0.26, 0.50]	0	< 0.001
Duration of mechanical ventilation	4	639	Inverse variance, random	−31.87 [−54.26, −9.49]	91	0.005
Length of stay in the ICU	4	497	Inverse variance, random	−3.10 [−6.35, 0.14]	96	0.06
Patient satisfaction	3	553	Mantel-Haenszel, random	1.16 [1.10, 1.24]	0	< 0.001
Patient agitated or anxiety rate	4	463	Mantel-Haenszel, random	0.68 [0.09, 5.22]	92	0.71

#### 3.4.2 Effect of guide-based interventions on the delirium incidence and duration of delirium

Six studies (19, 24, 28–30, 34) of 14 studies assessed the impact of guide-based interventions on the delirium incidence among ICU patients. Using a fixed-effects model, the analysis revealed that the delirium incidence in the experimental group was 0.53 times than that in the control group (RR = 0.53, 95% CI 0.41–0.68, *P* < 0.001, *I*^2^ = 0%). Additionally, three studies (19, 24, 28) of 14 studies assessed the impact of guide-based interventions on the duration of delirium among ICU patients. A random-effects model demonstrated that guide-based interventions significantly reduced the duration of delirium compared to control groups (WMD = −11.94, 95% CI −20.75 to −3.13, *P* = 0.008, *I*^2^ = 89%; Table 3, Supplementary Figures S1, S2). Furthermore, Tao et al.'s (28) study found that guide-based interventions delayed the onset time of delirium (2.31 ± 0.67 vs. 2.98 ± 0.72, *P* < 0.05) compared to control groups.

#### 3.4.3 Effect of guide-based interventions on the unplanned extubation rate and the other complications rate

Ten studies (20, 26–33, 35) of 14 studies assessed the impact of guide-based interventions on the unplanned extubation rate among ICU patients. Using a fixed-effects model, the analysis showed that the unplanned extubation rate in the experimental group was 0.36 times than that in the control group (RR = 0.36, 95% CI 0.23 to 0.56, *P* < 0.001; *I*^2^ = 0%). Additionally, nine studies (20, 26, 27, 29–33, 35) of 14 studies assessed the impact of guide-based interventions on the other complications rate among ICU patients. The fixed-effects model indicated that the other complications rate in the experimental group was 0.36 times than that in the control group (RR = 0.36, 95% CI 0.26–0.50, *P* < 0.001; *I*^2^ = 0%; Table 3, Supplementary Figures S3, S4).

#### 3.4.4 Effect of guide-based interventions on the duration of mechanical ventilation and length of stay in the ICU

Four studies (19, 24, 28, 34) of 14 studies assessed the impact of guide-based interventions on the duration of mechanical ventilation among ICU patients. A random-effects model revealed that guide-based interventions significantly reduced the duration of mechanical ventilation compared to control groups (WMD = −31.87, 95% CI: −54.26 to −9.49, *P* = 0.005, *I*^2^ = 91%). Additionally, three studies (19, 24, 28, 34) of 14 studies assessed the impact of guide-based interventions on the length of stay in the ICU. The random-effects model indicated no significant difference in the length of stay in the ICU between the experimental and control groups (WMD = −3.1, 95% CI: −6.35 to 0.14, *P* = 0.06, *I*^2^ = 96%; Table 3, Supplementary Figures S5, S6).

#### 3.4.5 Effect of guide-based interventions on the patient satisfaction and patient agitated or anxiety rate

Three studies (27, 31, 35) of 14 studies assessed the impact of guide-based interventions on patient satisfaction. Using a random-effects model, the analysis revealed that patient satisfaction in the experimental group was 1.16 times than that in the control group (RR = 1.16, 95% CI 1.10–1.24, *P* < 0.001; *I*^2^ = 0%). It significantly improved patient satisfaction, representing a key positive outcome in terms of patient experience. Additionally, four studies (26, 27, 30, 32) of 14 studies assessed the impact of guide-based interventions on patient agitation or anxiety rates. The random-effects model indicated no significant difference in agitation or anxiety rates between the experimental and control groups (RR = 0.68, 95% CI 0.09–5.22, *P* = 0.71; *I*^2^ = 92%; Table 3, Supplementary Figures S7, S8).

### 3.5 Sensitivity analysis

A sensitivity analysis was performed on studies demonstrating significant heterogeneity *(P* < 0.01, *I*^2^ > 50%). Upon conducting a leave-one-out analysis, the heterogeneity of the remaining studies decreased (*I*^2^ < 50%). Nonetheless, the PR time results were altered *(P* > 0.05; refer to Table 4). The sensitivity analyses indicated increased heterogeneity, primarily attributed to variations in sample size and the quality of the literature, with a focus on these particular studies (25, 28) (Table 4).

**Table 4:** Sensitivity analysis of included studies.

**Outcomes**	***I*^2^ value (%) before**	**Number of excluded studies**	**Statistical method**	**Effect estimate**	***I*^2^ value (%) after**	***P-*value**
PR rate	95	3 [35, 39, 40]	Mantel-Haenszel, fixed	0.70 [0.65, 0.75]	14	< 0.001
PR time	89.8	3 [35, 41, 45]	Inverse variance, fixed	−49.54 [−86.92, −12.17]	58	0.12
Duration of delirium	89	1 [38]	Inverse variance, fixed	−5.76 [−6.64, −4.88]	0	< 0.001
Duration of mechanical ventilation	91	2 [29, 38]	Inverse variance, fixed	−26.20 [−34.78, −17.62]	0	< 0.001

### 3.6 Analysis of publication bias

In this study, we constructed funnel plots to analyze the PR rate and the unplanned extubation rate. The funnel plot analysis revealed a relatively symmetrical distribution of effect sizes along the central axis, with most data points evenly dispersed on both sides of the plot, indicating a low likelihood of significant publication bias in the included studies. This is further supported by the Begg and Egger tests, which showed *P* > 0.05 for unplanned extubation rate (Supplementary Figure S9). However, evidence of publication bias was detected for the PR rate, necessitating cautious interpretation (Egger test, *P* = 0.004; Supplementary Figure S10). To further validate the stability of our findings, we performed a supplementary analysis using the trim-and-fill method to assess potential publication bias. The comparative analysis demonstrated consistent effect estimates between pre- and post-adjustment results, with no significant alterations in the direction or magnitude of the observed effects. This methodological validation confirms the stability and reliability of the pooled effect size, suggesting that our primary findings are not substantially influenced by potential publication bias (Supplementary Figure S11, Supplementary Table S1).

## 4 Discussion

### 4.1 Discussion of the main findings

This study included 14 RCTs with 4,338 ICU patients to assess guide-based interventions for reducing the utilization of PR. While most studies couldn't achieve strict double-blindness due to the nature of the interventions, their overall quality is fair with clinical relevance. Main findings indicate that guide-based interventions can reduce the utilization of PR, delirium incidence and duration, unplanned extubation, other complications, and mechanical ventilation duration, while improving patient satisfaction. However, they don't shorten the length of stay in the ICU or improve patient agitation or anxiety. Multi-center and larger RCTs are needed for further validation.

In the included 14 RCTs, the guide, for formulating a protocol to reduce the utilization of PR, includes CPGs, syntheses of best evidence, ABCDEF bundle and scoping review. The decision of PR with patients in ICU is often based on clinical experience, hence, the objectivity and standardization of PR decision-making are insufficient (39, 40). Thus, standardized assessment is crucial. Our findings indicate that the PR Decision Wheel and Assessment of PR framework is the most prevalent in different studies, with the corresponding interventions frequently exhibiting similarities. Most interventions have instituted PR decision-making teams (24, 28, 29, 31–33, 35). Assessment is essential for all intervention; however, the content and frequency of these assessments vary considerably. For comprehensive assessment of PR, certain studies implemented assessments at 8-h intervals (20, 27), whereas others implemented assessments at the conclusion of each shift, and the necessity for PR is re-evaluated within the same timeframe (30, 32). This meta-analysis specifically evaluated the effects of guide-based interventions on reducing the utilization of PR with patients in ICU. Firstly, this study found that guide-based interventions significantly reduce the utilization of PR with ICU patients. Interventions grounded in the PR Decision Wheel and the Assessment of PR entail the selection of diverse PR methodologies tailored to the severity of the patient's condition, with adjustments to the frequency of assessments as necessary (20, 26, 32). This approach has demonstrated efficacy in reducing the utilization of PR. Interventions based on the ABCEDEF bundle have demonstrated potential in reducing the utilization of PR (24, 28, 34). The bundle, however, consists of a multitude of interventions, incorporating diverse elements such as education and training (41), policy development (42), environmental modifications (43), and interdisciplinary collaboration (44). This complexity poses a challenge in determining the effectiveness of each individual component. Thus, additional research with factorial designs may be needed to identify the bundle's most effective components. While objective tools are available, their effective utilization by nurses necessitates a shift in their perception of PR. Research examining the perspectives of nursing staff on PR utilization reveals that, despite an awareness of its negative implications, entrenched practices and institutional norms frequently sustain its application (45, 46). This suggests a complex interaction between knowledge and practice, wherein even well-informed staff may encounter challenges in effectively implementing PR-reduction interventions due to systemic pressures and resource constraints. Consequently, numerous studies incorporate training programs for nurses to support this transition. Critical care nurses, who play a pivotal role in decision-making regarding the implementation of PR in ICUs. Through comprehensive training and the provision of clear guidelines, nurses can be better prepared to make informed decisions regarding the adjustment of assessment frequency as needed, potentially reducing reliance on these measures (45). Interventions based on syntheses of best evidence can be implemented to enhance nurses' decision-making skills, which has also been shown to effectively reduce the utilization of PR (25, 30). While this study provides evidence that guide-based interventions significantly reduce PR time, substantial heterogeneity was observed across studies. Subsequent subgroup analysis based on different PR time calculation methodologies revealed a marked reduction in heterogeneity, indicating that variations in time measurement formulas significantly influence the reported PR time outcomes. This finding suggests that standardization of PR time calculation methods is crucial for ensuring consistency and comparability across future studies in this field. Once the method for calculating PR time is standardized, all research findings will be based on a unified evaluative framework. This will enable researchers to clearly compare the core conclusions of different studies, accurately identify common patterns and distinct issues within the research, significantly enhance the efficiency of research utilization, and increase the academic value of the findings. Moreover, it will provide robust support for the transmission of knowledge and foster innovative breakthroughs within the field. Delirium represents a prevalent neuropsychiatric syndrome in ICU, demonstrating significant associations with multiple adverse clinical outcomes (11). PR has been identified as a modifiable risk factor and potential precipitant for delirium development (13). Therefore, reducing the utilization of PR is an important measure to prevent delirium from occurring. In our meta-analysis, we found that guide-based interventions were effective in reducing delirium incidence (19, 30, 34) and duration of delirium (19, 24, 28) and delaying delirium onset time (28). Guide-based interventions encompass the reduction of stress (e.g., family support, encourage preferences and identify and solve spiritual, social and environmental needs), the promotion of early activity (e.g., full range of joint motion, sitting exercise, bed-free activities, and walking exercises) and the titration of sedative and analgesic medications in accordance with the Richmond Agitation-Sedation Scale (RASS). Moreover, a study underscores the psychological effects of PR on family members during the COVID-19 pandemic, highlighting that involving them in care discussions and decisions can facilitate their understanding and coping with the utilization of PR, ultimately contributing to its reduction (47). Our study indicated that guide-based interventions can also shorten duration of mechanical ventilation. Guide-based interventions involve the systematic and timely assessment of pain, as well as the prompt removal of tubes. The reduction in the utilization of PR may contribute to a shortened duration of mechanical ventilation; however, this outcome is substantially affected by the patient's clinical condition. In clinical practice, it is imperative to develop a comprehensive, multi-dimensional evaluation framework. This framework should encompass, firstly, an assessment of individual patient variability, including factors such as age and history of underlying diseases, to ascertain the patient's tolerance to analgesic medications. Secondly, it should involve a thorough review of the intervention's implementation details, such as the precision of pain assessments and adherence to established protocols for the timing of tube removal. Concurrently, there is a need for dynamic monitoring of adverse reactions during the use of PR, integrating objective data to evaluate the feasibility and potential risks associated with reducing PR use. Therefore, a thorough evaluation of all relevant factors is imperative.

While PR is considered a preventive measure for unplanned extubation, relevant studies have pointed out that the longer the PR time, the greater the probability of unplanned extubation and the other complications rate also increases (13, 48). Meanwhile, high-quality meta-analyses have demonstrated that PR is an independent risk factor for unplanned extubation in ICU patients (49). The results of this study showed that the guide-based interventions can reduce unplanned extubation with patient in ICU. Guide-based interventions encompass the education and training regarding the knowledge of unplanned extubation, along with continuous assessment to ensure the timely removal of tubes. Interventions designed to minimize the application of PR may also be employed to reduce the incidence of unplanned extubations. Implementing these measures not only improve nursing quality but also enhance patient safety. In recent years, with the increasing attention of nursing managers to the systematic and normalized special training of nurses, the awareness of ICU nurses on PR has been strengthened. ICU nurses with strong PR awareness can accurately judge the timing and extent of PR reduction by dynamically assessing the patient's condition and the unplanned extubation rate has also decreased compared with the previous in routine care. Reduced utilization of PR can control the incidence of adverse events. Guide-based interventions are capable of evaluating the patient's PR level, timing, and release schedule through dynamic assessment. This approach can effectively minimize unnecessary PR and enhance the rationality and standardization of PR practices.

Based on the results of the meta-analysis, implementing the PR-reduction protocol in accordance with established guides and employing a multidisciplinary team approach are possible to reduce the utilization of PR. The frequency of patient assessments should be adjusted based on the specific type of PR involved. However, the variability in measurement tools, such as the PR Decision Wheel, RASS, CAM-ICU, and self-compiled ICU Patient PR Assessment Scale, can compromise the scientific rigor of the research. Consequently, the findings may be challenging to generalize. Therefore, there is a need to standardize the PR assessment scale.

### 4.2 Future expectations

Guide-based interventions show benefits in reducing the utilization of PR with patients in ICU. However, due to the diversity of guide-based interventions, determining the most effective guide-based protocol remains challenging. Further research utilizing factorial designs may be necessary to ascertain the most effective components of the guide. Furthermore, this study did not demonstrate improvement in patient agitation or anxiety and reduction length of stay in the ICU with guide-based intervention. Multi-center and larger RCTs are needed to validate these outcomes.

## 5 Limitation

The present review is subject to several limitations that warrant consideration. First, not all studies were included in each outcome analysis, which may affect the aggregated results and contribute to increased heterogeneity. Nonetheless, we meticulously examined the full text to minimize data loss. Second, despite our thorough analysis and synthesis of the guide-based interventions described across all studies, inherent differences among these interventions may unavoidably enhance heterogeneity in the findings, necessitating further detailed investigation. Third, a potential source of bias may arise and the generalizability may be constrained from the geographical concentration of the studies, as 13 out of the 14 studies analyzed were conducted in China. Cultural norms, ethics, and healthcare systems significantly shape practices and attitudes toward PR. In China, familism and medical paternalism may lead to more acceptance of PR for safety, contrasting with Western emphasis on individual autonomy. Thus, caution is needed when applying these findings to other cultural contexts. Future research should include diverse populations globally to assess the general applicability of these interventions. Fourth, three of the studies included in the analysis were guided by the ABCDEF bundle. Of these, two studies focused specifically on the early mobility and exercise components of the intervention package. In contrast, the third study implemented a comprehensive, multi-layered ABCDEF bundle, which obscured the identification of the specific elements responsible for the observed effects. Therefore, further research utilizing factorial designs may be necessary to ascertain the most effective components of the bundle. Finally, it is important to note that the current evidence is single-center studies with relatively small sample sizes among the 14 included articles. To strengthen the validity and generalizability of these findings, future research should prioritize the implementation of multicenter, large-scale RCTs.

## 6 Conclusion

Guide-based interventions can effectively reduce the utilization of PR with patients in ICU. Employing a multidisciplinary team and adjusting patient assessment frequency based on the type of PR are efficient. Meanwhile, it is recommended to standardize the patient PR assessment scale to enhance the comparability of study results and the precision of treatment. Additionally, the evidence from this meta-analysis suggests that guide-based interventions show promise in decreasing delirium, unplanned extubation rate, the other complications rate, and enhancing patient satisfaction. Given the relatively limited sample size of included studies in the current review, there is a need for future research to incorporate more RCTs that implement carefully designed, guide-based intervention protocols.

## Data Availability

The original contributions presented in the study are included in the article/Supplementary material, further inquiries can be directed to the corresponding authors.
