# Supplementary material for: Guide-based interventions aimed at reducing physical restraints in intensive care unit: a systematic review and meta-analysis of randomized controlled trials
Source: Front Med (Lausanne). 2025 Sep 26;12:1606359. doi: 10.3389/fmed.2025.1606359 (PMC12511021; doi:10.3389/fmed.2025.1606359)
Supplement: Supplementary file 1 [file Supplementary_file_1.docx]

**Appendix. Search Strategy**

**1、CNKI**

①主题：（身体约束 + 保护性约束 + 限制性约束 + 物理约束 + 约束 + 约束带 + 约束手套 + 约束衣）（427，886）

②主题：（重症监护 + 重症医学科 + 加强监护病房 + ICU + EICU + SICU + CCU） （89，523）

③主题：（临床实践指南 + 实践指南 + 诊疗指南 + 指南 + 诊疗指导 + 指导方针 + 诊疗准则） （109，576）

④主题：（证据 + 循证 + 循证医学 + 循证护理 + 循证实践 + EBM + EBP + EBN） （241，944）

⑤主题：（集束化 + 治疗方案 + 治疗路径 + 临床方案 + 临床路径 + 护理清单 + 检查清单） （115，951）

⑥ ③ or ④ or ⑤（456,030）

⑦ ① and ② and ⑥ （78）

**2、Wanfang、VIP**

①题名或关键词: (身体约束 or 保护性约束 or 限制性约束 or 物理约束 or 约束 or 约束带 or 约束手套 or 约束衣) （维普110,272）(万方 177,322)

②题名或关键词: (重症监护 or 重症医学科 or 加强监护病房 or ICU or EICU or SICU or CCU) （维普62,166）(万方103,070)

③题名或关键词：（临床实践指南 or 实践指南 or 诊疗指南 or 指南 or 诊疗指导 or 指导方针 or 诊疗准则）（维普92,370）(万方133,208)

④题名或关键词：（证据 or 循证 or 循证医学 or 循证护理 or 循证实践 or EBM or EBP or EBN）（维普116,437）(万方162,051)

⑤题名或关键词：（集束化 or 治疗方案 or 治疗路径 or 临床方案 or 临床路径 or 护理清单 or 检查清单）（维普77,543）(万方56,828)

⑥ ③ or ④ or ⑤ （维普283,150）(万方348,812)

⑦ ① and ② and ⑥ （维普29）(万方31)

**3、SinoMed**

#1 "约束, 身体的"[不加权:扩展] **（638）**

#2 "身体约束"[核心字段:智能] OR "保护性约束"[核心字段:智能] OR "限制性约束"[核心字段:智能] OR "物理约束"[核心字段:智能] OR "约束"[核心字段:智能] OR "约束带"[核心字段:智能] OR "约束手套"[核心字段:智能] OR "约束衣"[核 心字段:智能] **（2651）**

#3 (#2) OR (#1) **（2651）**

#4 "重症监护病房"[不加权:扩展]) OR "危重病人医疗"[不加权:扩展]) OR "危重症护理"[不加权:扩展]) OR "危重症监护结局"[不加权:扩展]) OR "危重病人医疗"[不加权:扩展] **（62836）**

#5 "重症监护"[核心字段:智能] OR "重症医学科"[核心字段:智能] OR "加强监护病房"[核心字段:智能] OR "ICU"[核心字段:智能] OR "EICU"[核心字段:智能] OR "SICU"[核心字段:智能] OR "CCU"[核心字段:智能]) **（64379）**

#6 (#5) OR (#4) **（65277）**

#7 "指导方针(主题)"[不加权:扩展]) OR "诊疗准则"[不加权:扩展] OR "诊疗准则(主题)"[不加权:扩展]) OR "坚持准则"[不加权:扩展]) OR "指南"[不加权:扩展] OR "诊疗指南"[不加权:扩展] **（3573）**

#8 "临床实践指南"[核心字段:智能] OR "实践指南"[核心字段:智能] **（2284）**

#9  [(#8) OR (#7)](javascript:toDoRelimitSearch();) **（5683）**

#10 "循证护理学"[不加权:扩展]) OR "循证实践"[不加权:扩展]) OR "循证医学"[不加权:扩展]) OR "循证急救医学"[不加权:扩展] **（26440）**

#11 "证据"[核心字段:智能] OR "循证"[核心字段:智能] OR "EBP"[核心字段:智能] OR "EBM"[核心字段:智能] OR "EBN"[核心字段:智能] **（35551）**

#12 (#11) OR (#10) **（35551）**

#13 "病人护理包"[不加权:扩展]) OR "临床路径"[不加权:扩展]) OR "临床方案"[不加权:扩展]  **(27876)**

#14 "集束化"[核心字段:智能] OR "护理清单"[核心字段:智能] OR "检查清单"[核心字段:智能] OR "治疗方案"[核心字段:智能] OR "治疗路径"[核心字段:智能] **（32236）**

#15 (#14) OR (#13) **（42781）**

#16 (#15) OR (#12) OR (#9) **（83063）**

#17 (#16) AND (#6) AND (#3) **（27）**

**4、PubMed**

#1 Restraint, Physical [MeSH Major Topic] **（5537）**

#2 (physical restraint*[Title/Abstract]) OR (physical constraint*[Title/Abstract]) OR (mechanical restraint*[Title/Abstract]) OR (mechanical constraint*[Title/Abstract])**（4975）**

#3 (disallow* movement*[Title/Abstract]) OR (limit* movement*[Title/Abstract]) OR (restrict* movement*[Title/Abstract]) OR (stop* movement*[Title/Abstract])**（1577）**

#4 ((ankle*[Title/Abstract]) OR (appendage*[Title/Abstract]) OR (arm*[Title/Abstract]) OR (body[Title/Abstract]) OR (bodies[Title/Abstract]) OR (foot[Title/Abstract]) OR (feet[Title/Abstract]) OR (hand*[Title/Abstract]) OR (leg*[Title/Abstract]) OR (limb*[Title/Abstract]) OR (patient*[Title/Abstract]) OR (wrist*[Title/Abstract])) AND ((tie*[Title/Abstract]) OR (bedrail*[Title/Abstract]) OR (belt*[Title/Abstract]) OR (strap*[Title/Abstract]) OR (mitten*[Title/Abstract]) OR (jacket*[Title/Abstract]))**（29,432）**

#5 #1 OR #2 OR #3 OR #4 **（40,206）**

#6 Intensive Care Units [MeSH Major Topic] **（47,403）**

#7 Critical Care [MeSH Major Topic] **（39,026）**

#8 Critical Care Nursing [MeSH Major Topic] **（2190）**

#9 (intensive care[Title/Abstract]) OR (critical care[Title/Abstract]) OR (acute care[Title/Abstract])**（272,812）**

#10 ((cardiac[Title/Abstract]) OR (coronary[Title/Abstract]) OR (heart[Title/Abstract]) OR (burn[Title/Abstract]) OR (respiratory[Title/Abstract]) OR (surgical[Title/Abstract]) OR (surger*[Title/Abstract]) OR (high dependency[Title/Abstract]) OR (stepdown[Title/Abstract])OR (step-down[Title/Abstract]) OR (speciali#ed weaning[Title/Abstract]) OR (postoperati*[Title/Abstract]) OR (postoperati*[Title/Abstract]) OR (postsurg*[Title/Abstract]) OR (post-surg*[Title/Abstract])) AND ((unit*[Title/Abstract]) OR (centre*[Title/Abstract]) OR (center*[Title/Abstract]))**（1,477,916）**

#11 (ICU*[Title/Abstract]) OR (SICU*[Title/Abstract]) OR (CCU*[Title/Abstract]) OR (EICU*[Title/Abstract]) OR (HDU*[Title/Abstract]) OR (SDU*[Title/Abstract]) OR (EDSDU*[Title/Abstract]) **（99,361）**

#12 #6 OR #7 OR #8 OR #9 OR #10 OR #11 **（1,691,663）**

#13 Guidelines as Topic[MeSH Major Topic] (60,655)

#14 Practice Guidelines as Topic[MeSH Major Topic]（46,804）

#15 Guideline Adherence[MeSH Major Topic]（17,203）

#16 ((clinical[Title/Abstract] OR clinic[Title/Abstract] OR health[Title/Abstract] OR practice[Title/Abstract] OR diagnostic[Title/Abstract] OR diagnosis[Title/Abstract] OR therapy[Title/Abstract] OR therapies[Title/Abstract])) AND ((guideline*[Title/Abstract] OR guidance[Title/Abstract] OR recommendation[Title/Abstract] OR recommendations[Title/Abstract] OR standard*[Title/Abstract] OR pathway*[Title/Abstract] OR protocol*[Title/Abstract])) (1,993,509)

#17 ((guideline*[Title/Abstract] OR guidance[Title/Abstract] OR standard*[Title/Abstract] OR pathway*[Title/Abstract] OR protocol*[Title/Abstract])) AND ((implement*[Title/Abstract] OR uptake*[Title/Abstract] OR adopt*[Title/Abstract] OR adhere*[Title/Abstract] OR concord*[Title/Abstract] OR complian*[Title/Abstract] OR comply[Title/Abstract] OR non-adhere*[Title/Abstract] OR nonadhere*[Title/Abstract] OR non-concord*[Title/Abstract] OR nonconcord*[Title/Abstract] OR non-complian*[Title/Abstract] OR noncomplian*[Title/Abstract])) (477,951)

#18 #13 OR #14 OR #15 OR #16 OR #17 (2,217,862)

#19 Evidence-Based Nursing[MeSH Major Topic] (2509)

#20 Evidence-Based Practice[MeSH Major Topic] （36,336）

#21 Evidence-Based Medicine[MeSH Major Topic] (27,456)

#22 Evidence-Based Emergency Medicine[MeSH Major Topic] (169)

#23 ((evidence[Title/Abstract] OR evidence-based[Title/Abstract] OR EBM[Title/Abstract] OR EBN[Title/Abstract] OR EBP[Title/Abstract] OR best practice*[Title/Abstract] OR knowledge[Title/Abstract])) AND ((care[Title/Abstract] OR healthcare[Title/Abstract] OR nurs*[Title/Abstract] OR patient care[Title/Abstract] OR practice?[Title/Abstract] OR practitioner?[Title/Abstract])) (605,239)

#24 ((evidence[Title/Abstract] OR evidence-based[Title/Abstract] OR EBM[Title/Abstract] OR EBN[Title/Abstract] OR EBP[Title/Abstract] OR best practice*[Title/Abstract] OR knowledge[Title/Abstract])) AND ((adopt*[Title/Abstract] OR application[Title/Abstract] OR apply*[Title/Abstract] OR diffusion[Title/Abstract] OR implement*[Title/Abstract] OR uptake*[Title/Abstract] OR transfer*[Title/Abstract] OR translat*[Title/Abstract] OR utili?ation[Title/Abstract] OR utili?e?[Title/Abstract] OR utili?ing[Title/Abstract])) (563,346)

#25 #19 OR #20 OR #21 OR #22 OR #23 OR #24 (1,010,748)

#26 Critical Pathways[MeSH Major Topic]**（4577）**

#27 Clinical Protocols[MeSH Major Topic] **（108,899）**

#28 Patient Care Bundles[MeSH Major Topic] **（1059）**

#29 ((care[Title/Abstract] OR evidence[Title/Abstract] OR treatment[Title/Abstract] OR clinical[Title/Abstract] OR critical[Title/Abstract] OR health[Title/Abstract] OR practice[Title/Abstract])) AND ((package*[Title/Abstract] OR checklist*[Title/Abstract] OR check list[Title/Abstract] OR algorithm[Title/Abstract] OR bundl*[Title/Abstract] OR map*[Title/Abstract] OR path[Title/Abstract] OR paths[Title/Abstract] OR pathway*[Title/Abstract] OR protocol*[Title/Abstract])) (1,388,545)

#30 #26 OR #27 OR #28 OR #29 (1,485,529)

#31 #30 OR #25 OR #18 (3,675,844)

#32 #31 AND #12 AND #5 (763)

**5、****Cochrane library**

#1 MeSH descriptor: [Restraint, Physical] explode all trees (324)

#2 ((physical or mechanical) near (constraint* or restraint*)): ti,ab,kw (713)

#3 ((disallow* or limit* or restrict* or stop*) near (movement*)): ti,ab,kw (1766)

#4 ((ankle* or appendage* or arm* or body or bodies or foot or feet or hand* or leg* or limb* or patient* or wrist*) near (tie* or bedrail* or belt* or strap* or mitten* or jacket*)): ti,ab,kw (1200)

#5 #1 OR #2 OR #3 OR #4 (3654)

#6 MeSH descriptor: [Intensive Care Units] explode all trees (6247)

#7 MeSH descriptor: [Critical Care] explode all trees (3117)

#8 MeSH descriptor: [Critical Care Nursing] explode all trees (80)

#9 ((intensive or critical or acute) near (care)): ti,ab,kw (43829)

#10 ((cardiac or coronary or heart or burn or respiratory or surgical or surger* or high dependency or stepdown or step-down or speciali#ed weaning or postoperati* or post-operati* or postsurg* or post-surg*) near (unit* or centre* or center*)): ti,ab,kw (22967)

#11 (ICU* or SICU* or CCU* or EICU* or HDU* or SDU* or EDSDU*):ti,ab,kw (21393)

#12 #6 OR #7 OR #8 OR #9 OR #10 OR #11 (70171)

#13 MeSH descriptor: [Guidelines as Topic] explode all trees (3816)

#14 MeSH descriptor: [Practice Guidelines as Topic] explode all trees（3200）

#15 MeSH descriptor: [Guideline Adherence] explode all trees（1619）

#16 ((clinical or clinic or health or practice or diagnostic or diagnosis or therapy or therapies) near (guideline* or guidance or recommendation or recommendations or standard* or pathway* or protocol*)):ti,ab,kw (123283)

#17 ((guideline* or guidance or standard* or pathway* or protocol*) near (implement* or uptake* or adopt* or adhere* or concord* or complian* or comply or non-adhere* or nonadhere* or non-concord* or nonconcord* or non-complian* or noncomplian*)):ti,ab,kw (23384)

#18 #13 OR #14 OR #15 OR #16 OR #17 (138012)

#19 MeSH descriptor: [Evidence-Based Nursing] explode all trees (94)

#20 MeSH descriptor: [Evidence-Based Practice] explode all trees（4024）

#21 MeSH descriptor: [Evidence-Based Medicine] explode all trees (3383)

#22 MeSH descriptor: [Evidence-Based Emergency Medicine] explode all trees (6)

#23 ((evidence or evidence-based or EBM or EBN or EBP or best practice* or knowledge) near (care or healthcare or nurs* or patient care or practice? or practitioner?)):ti,ab,kw (159179)

#24 ((evidence or evidence-based or EBM or EBN or EBP or best practice* or knowledge) near (adopt* or application or apply* or diffusion or implement* or uptake* or transfer* or translat* or utili?ation or utili?e? or utili?ing)):ti,ab,kw (16009)

#25 #19 OR #20 OR #21 OR #22 OR #23 OR #24 (165226)

#26 MeSH descriptor: [Patient Care Bundles] explode all trees **（78）**

#27 MeSH descriptor: [Critical Pathways] explode all trees**（329）**

#28 MeSH descriptor: [Clinical Protocols] explode all trees **（28183）**

#29 ((care or evidence or treatment or clinical or critical) near (package* or checklist* or check list* or algorithm* or bundl* or map* or path or paths or pathway* or protocol*)):ti,ab,kw (61963)

#30 #26 OR #27 OR #28 OR #29 (83297)

#31 #30 OR #25 OR #18 (303823)

#32 #31 AND #12 AND #5 (85)

Review 7 Trials 78

**6、****Web of Science**

#1 TS=((physical OR mechanical) NEAR/2 (constraint* OR restraint*)) (37,797)

#2 TS=((ankle* OR appendage* OR arm* OR body OR bodies OR foot OR feet OR hand* OR leg* OR limb* OR patient* OR wrist*) NEAR/2 (tie* OR bedrail* OR belt* OR strap* OR mitten* OR jacket*)) (177,141)

#3 #1 OR #2 (214,870)

#4 TS=(intensive care unit OR intensive care OR intensive care nursing) (415,306)

#5 TS=((intensive OR critical OR acute) NEAR/2 (care)) (496,355)

#6 TS=((cardiac OR coronary OR heart OR burn OR respiratory OR surgical OR surger* OR high-dependency OR stepdown OR speciali?ed-weaning OR postoperati* OR postsurg*) NEAR/2 (unit* OR centre* OR center*)) (104,225)

#7 TS=(icu* OR sicu* OR ccu* OR eicu* OR hdu* OR sdu* OR edsdu*) (174,837)

#8 #4 OR #5 OR #6 OR #7 (681,149)

#9 TS=(guideline adherence OR practice guidelines) (429,087)

#10 TS=((clinical or clinic or health or practice or diagnostic or diagnosis or therapy or therapies) NEAR/2 (guideline* or guidance or recommendation or recommendations or standard* or pathway* or protocol*)) (679,954)

#11 TS=((guideline* or guidance or standard* or pathway* or protocol*) NEAR/2 (implement* or uptake* or adopt* or adhere* or concord* or complian* or comply or non-adhere* or nonadhere* or non-concord* or nonconcord* or non-complian* or noncomplian*)) (228,834)

#12 #9 OR #10 OR #11 (1,018,770)

#13 TS=(Evidence-Based Nursing OR Evidence-Based Practice OR Evidence-Based Medicine OR Evidence-Based Emergency Medicine) (276,527)

#14 TS=((evidence or evidence-based or EBM or EBN or EBP or "best practice*" or knowledge) NEAR/2 (care or healthcare or nurs* or "patient care" or practice? or practitioner?)) (199,988)

#15 TS=((evidence or evidence-based or EBM or EBN or EBP or "best practice*" or knowledge) NEAR/2 (adopt* or application or apply* or diffusion or implement* or uptake* or transfer* or translat* or utili?ation or utili?e? or utili?ing)) (659,991)

#16 #13 OR #14 OR #15 (168359)

#17 TS=(patient care bundles OR critical pathways OR clinical protocols) (901,311)

#18 TS=((care OR evidence OR treatment OR clinical OR critical) NEAR/3 (package* OR checklist* OR "check list*" OR algorithm* OR bundl* OR map* OR path OR paths OR pathway* OR protocol*)) (364,850)

#19#17 OR #18 (1,101,734)

#20 #12 OR #16 OR #19 (2,503,726)

#21 #20 AND #8 AND #3 (340)

**7、****CINAHL**

S1 (MH "Restraint, Physical") （4790)

S2 AB ((physical or mechanical) N2 (constraint* or restraint*))  （1630）

S3 AB ((disallow* or limit* or restrict* or stop*) N2 (movement*))  （1881）

S4 AB ((ankle* or appendage* or arm* or body or bodies or foot or feet or hand* or leg* or limb* or patient* or wrist*) N2 (tie* or bedrail* or belt* or strap* or mitten* or jacket*)) （576）

S5 S1 OR S2 OR S3 OR S4  （7930）

S6 (MH "Intensive Care Units") OR (MH "Coronary Care Units") OR (MH "Post Anesthesia Care Units") OR (MH "Critical Care Nursing") OR (MH "Critical Care") OR (MH "Burn Units") OR (MH "Observation Units") （88，465）

S7 AB ((intensive or critical or acute) N2 (care))  （100，055）

S8 AB ((cardiac or coronary or heart or burn or respiratory or surgical or surger* or high dependency or stepdown or step-down or speciali#ed weaning or postoperati* or post-operati* or postsurg* or post-surg*) N2 (unit* or centre* or center*))  （17，449）

S9 AB (ICU* or SICU* or CCU* or EICU* or HDU* or SDU* or EDSDU*)  （34，896）

S10 S6 OR S7 OR S8 OR S9  （171，852）

S11 (MH "Practice Guidelines") OR (MH "Guideline Adherence") OR (MH "Education, Continuing+") OR (MH "Education, Interdisciplinary") （143,167））

S12 (MH "Critical Path") (6,180)

S13 AB ((clinical or clinic or health or practice or diagnostic or diagnosis or therapy or therapies) N2 (guideline* or guidance or recommendation or recommendations or standard* or pathway* or protocol*)) (91,065))

S14 AB ((guideline* or guidance or standard* or pathway* or protocol*) N2 (implement* or uptake* or adopt* or adhere* or concord* or complian* or comply or non-adhere* or nonadhere* or non-concord* or nonconcord* or non-complian* or noncomplian*)) (26,877)

S15 S11 OR S12 OR S13 OR S14 (242,029)

S16 (MH "Nursing Practice, Evidence-Based") OR (MH "Nursing Practice, Research-Based") (16,877)

S17 ( (MH "Nurses+") OR TI ( nurse or nurses or nursing ) OR MW ( nurse or nurses or nursing ) ) AND ( (MH "Professional Practice, Evidence-Based") OR (MH "Medical Practice, Evidence-Based") OR (MH "Occupational Therapy Practice, Evidence-Based") OR (MH "Physical Therapy Practice, Evidence-Based") OR (MH "Professional Practice, Research-Based") OR (MH "Medical Practice, Research-Based") OR (MH "Occupational Therapy Practice, Research-Based") OR (MH "Physical Therapy") (1,003,391)

S18 TI (evidence* N2 nurs*) OR AB (evidence* N2 nurs*) OR TI (EBP N2 nurs*) OR AB (EBP N2 nurs*) OR TI (EBM N2 nurs*) OR AB (EBM N2 nurs*) OR TI (EBN N2 nurs*) OR AB (EBN N2 nurs*) (5,704)

S19 S16 OR S17 OR S18 (1,003,781)

S20 (MH "Care Bundle") OR (MH "Patient Care") OR (MH " Bundle") OR(MH " critical pathways") OR (MH " clinical protocols")  （43,324）

S21 AB (care OR evidence OR treatment OR clinical OR critical) N3 (package* OR checklist* OR check list* OR algorithm* OR bundl* OR map* OR path OR paths OR pathway* OR protocol*)  （42,897）

S22 S20 OR S21  （85,509）

S23 S15 OR S19 OR S22 (1,250,122)

S24 S5 AND S10 AND S23 (391)

**8、****EMBASE**

#1 'physical restraint'/exp （1198）

#2 ((physical OR mechanical) NEAR/2 (constraint* OR restraint*)):ti,ab,kw （6478）

#3 ((ankle* OR appendage* OR arm* OR body OR bodies OR foot OR feet OR hand* OR leg* OR limb* OR patient* OR wrist*) NEAR/2 (tie* OR bedrail* OR belt* OR strap* OR mitten* OR jacket*)):ti,ab,kw（4684）

#4 #1 OR #2 OR #3 （11,570）

#5 'intensive care unit'/exp （333,940）

#6 'intensive care'/exp （963,729）

#7 'intensive care nursing'/exp （3692）

#8 ((intensive OR critical OR acute) NEAR/2 care):ti,ab,kw （422,689）

#9 ((cardiac OR coronary OR heart OR burn OR respiratory OR surgical OR surger* OR 'high dependency' OR stepdown OR 'step down' OR 'speciali?ed weaning' OR postoperati* OR 'post operati*' OR postsurg* OR 'post surg*') NEAR/2 (unit* OR centre* OR center*)):ti,ab,kw (69,709)

#10 icu*:ti,ab,kw OR sicu*:ti,ab,kw OR ccu*:ti,ab,kw OR eicu*:ti,ab,kw OR hdu*:ti,ab,kw OR sdu*:ti,ab,kw OR edsdu*:ti,ab,kw (204,663)

#11 #5 OR #6 OR #7 OR #8 OR #9 OR #10 (1,384,884)

#12 'practice guidelines'/exp (770,853)

#13 'guideline adherence'/exp (22,981)

#14 ((clinical OR clinic OR health OR practice OR diagnostic OR diagnosis OR therapy OR therapies) NEAR/2 (guideline* OR guidance OR recommendation OR recommendations OR standard* OR pathway* OR protocol*)):ti,ab,kw (356,630)

#15 ((guideline* OR guidance OR standard* OR pathway* OR protocol*) NEAR/2 (implement* OR uptake* OR adopt* OR adhere* OR concord* OR complian* OR comply OR 'non adhere*' OR nonadhere* OR 'non concord*' OR nonconcord* OR 'non complian*' OR noncomplian*)):ti,ab,kw (90,427)

#16 #12 OR #13 OR #14 OR #15 (1,097,242)

#17 'evidence-based nursing'/exp (4963)

#18 'evidence-based practice'/exp （2,001,214）

#19 'evidence-based medicine'/exp (1,909,802)

#20 'evidence-based emergency medicine'/exp (432)

#21 ((evidence OR 'evidence based' OR ebm OR ebn OR ebp OR 'best practice*' OR knowledge) NEAR/2 (care OR healthcare OR nurs* OR 'patient care' OR practice? OR practitioner?)):ti,ab,kw (81,155)

#22 ((evidence OR 'evidence based' OR ebm OR ebn OR ebp OR 'best practice*' OR knowledge) NEAR/2 (adopt* OR application OR apply* OR diffusion OR implement* OR uptake* OR transfer* OR translat* OR utili?ation OR utili?e? OR utili?ing)):ti,ab,kw (51,705)

#23 #17 OR #18 OR #19 OR #20 OR #21 OR #22 (2,094,902)

#24 'care bundle'/exp (2189)

#25 'clinical pathway'/exp (10,606)

#26 'clinical protocol'/exp (129,334)

#27 ((care OR evidence OR treatment OR clinical OR critical) NEAR/3 (package* OR checklist* OR 'check list*' OR algorithm* OR bundl* OR map* OR path OR paths OR pathway* OR protocol*)):ti,ab,kw (213,190)

#28 #24 or #25 or #26 or #27 (325,553)

#29 #16 or #23 or #28 (3,0978,310)

#30 #4 and #11 and #29 (306)

**9、JBI**

#1 restraint, physical/

#2 ((physical or mechanical) adj2 (constraint* or restraint*)).ab,kw,ti. (20)

#3 ((disallow* or limit* or restrict* or stop*) adj2 movement*).ab,kw,ti. （2）

#4 ((ankle* or appendage* or arm* or body or bodies or foot or feet or hand* or leg* or limb* or patient* or wrist*) adj2 (tie* or bedrail* or belt* or strap* or mitten* or jacket*)).ab,kw,ti.

#5 #1 or #2 or #3 or #4 (21)

#6 intensive care units/ (5)

#7 critical care/ (16)

#8 ((intensive or critical or acute) adj2 care).ab,kw,ti. (334)

#9 ((cardiac or coronary or heart or burn or respiratory or surgical or surger* or high dependency or stepdown or step-down or speciali#ed weaning or postoperati* or post-operati* or postsurg* or post-surg*) adj2 (unit* or centre* or center*)).ab,kw,ti. (24)

#10 (ICU* or SICU* or CCU* or EICU* or HDU* or SDU* or EDSDU*).ab,kw,ti. (63)

#11 #6 or #7 or #8 or #9 or #10 (360)

#12 guidelines as topic/

#13 practice guidelines as topic/

#14 guideline adherence/

#15 ((clinical or clinic or health or practice or diagnostic or diagnosis or therapy or therapies) adj2 (guideline* or guidance or recommendation or recommendations or standard* or pathway* or protocol*)).ab,kw,ti. (195)

#16 ((guideline* or guidance or standard* or pathway* or protocol*) adj2 (implement* or uptake* or adopt* or adhere* or concord* or complian* or comply or non-adhere* or nonadhere* or non-concord* or nonconcord* or non-complian* or noncomplian*)).ab,kw,ti. (21)

#17 #12 or #13 or #14 or #15 or #16 (210)

#18 evidence-based nursing/

#19 evidence-based practice/ (14)

#20 evidence-based medicine/ (1)

#21 evidence-based emergency medicine/

#22 ((evidence or evidence-based or EBM or EBN or EBP or best practice* or knowledge) adj2 (care or healthcare or nurs* or patient care or practice? or practitioner?)).ab,kw,ti. (243)

#23 ((evidence or evidence-based or EBM or EBN or EBP or best practice* or knowledge) adj2 (adopt* or application or apply* or diffusion or implement* or uptake* or transfer* or translat* or utili?ation or utili?e? or utili?ing)).ab,kw,ti. (76)

#24 #18 or #19 or #20 or #21 or #22 or #23 (282)

#25 patient care bundles/

#26 critical pathways/ (1)

#27 clinical protocols/

#28 ((care or evidence or treatment or clinical or critical) adj3 (package* or checklist* or check list* or algorithm* or bundl* or map* or path or paths or pathway* or protocol*)).ab,kw,ti. (286)

#29 #25 or #26 or #27 or #28 (286)

#30 #17 or #24 or #29 (662)

#31 #5 and #11 and #30 （3）
