# Supplementary material for: Guide-based interventions aimed at reducing physical restraints in intensive care unit: a systematic review and meta-analysis of randomized controlled trials
Source: Front Med (Lausanne). 2025 Sep 26;12:1606359. doi: 10.3389/fmed.2025.1606359 (PMC12511021; doi:10.3389/fmed.2025.1606359)
Supplement: Supplementary file 2 [file Supplementary_file_2.docx]

**Appendix B.**

**Supplementary Figure 1:** Effect of guide-based interventions on the delirium incidence with patients in ICU.

**Supplementary Figure 2:** Effect of guide-based interventions on the duration of delirium with patients in ICU.

**Supplementary Figure 3:** Effect of guide-based interventions on the unplanned extubation rate with patients in ICU.

**Supplementary Figure 4:** Effect of guide-based interventions on the other complications rate with patients in ICU.

**Supplementary Figure 5:** Effect of guide-based interventions on the duration of mechanical ventilation with patients in ICU.

**Supplementary Figure 6:** Effect of guide-based interventions on the length of stay in the ICU with patients.

**Supplementary Figure 7:** Effect of guide-based interventions on the patient satisfaction with patients in ICU.

**Supplementary Figure 8:** Effect of guide-based interventions on the patient agitated or anxiety rate with patients in ICU.

**Supplementary Figure 9:** The funnel plot of unplanned extubation rate

**Supplementary Figure 10:** The funnel plot of PR rate

**Supplementary Figure 11:** The funnel plot of the incidence of PR rate

**Supplementary Table 1:** Effects of the incidence of PR rate by trim-and fill method


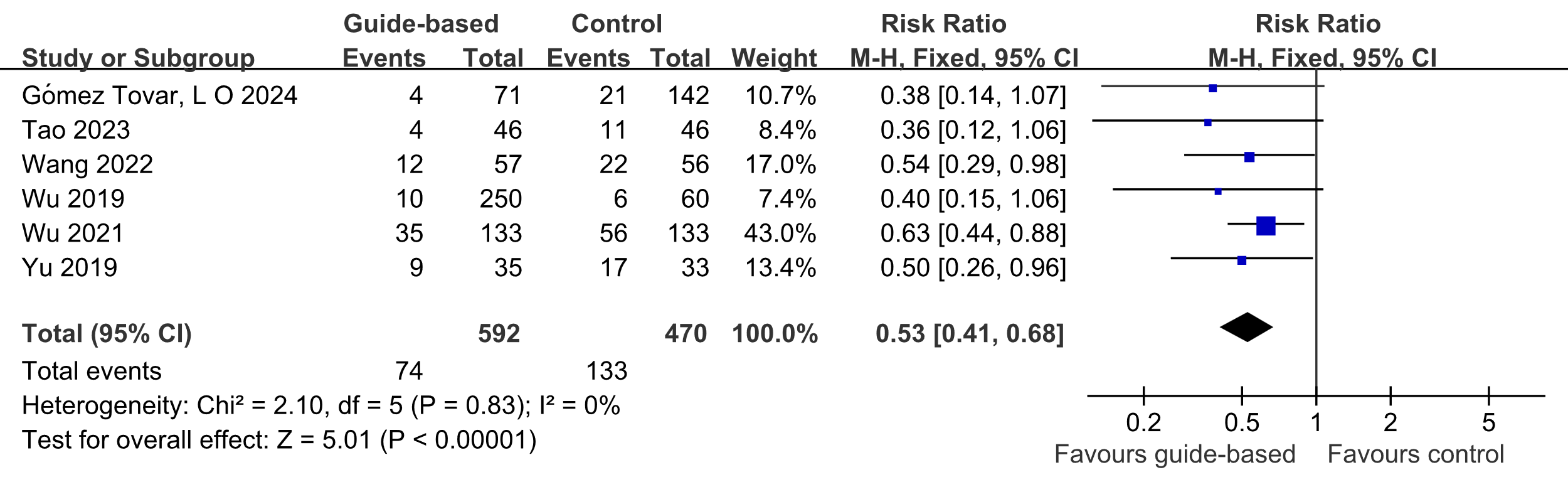


**Supplementary Figure 1: Effect of guide-based interventions on the delirium incidence with patients in ICU.**

When compared with control groups (*n* = 470), the guide-based intervention groups (*n* = 592) decreased the delirium incidence (RR=0.53, 95% CI 0.41 to 0.68, P＜0.001, I2=0%; ﬁxed-effects model).


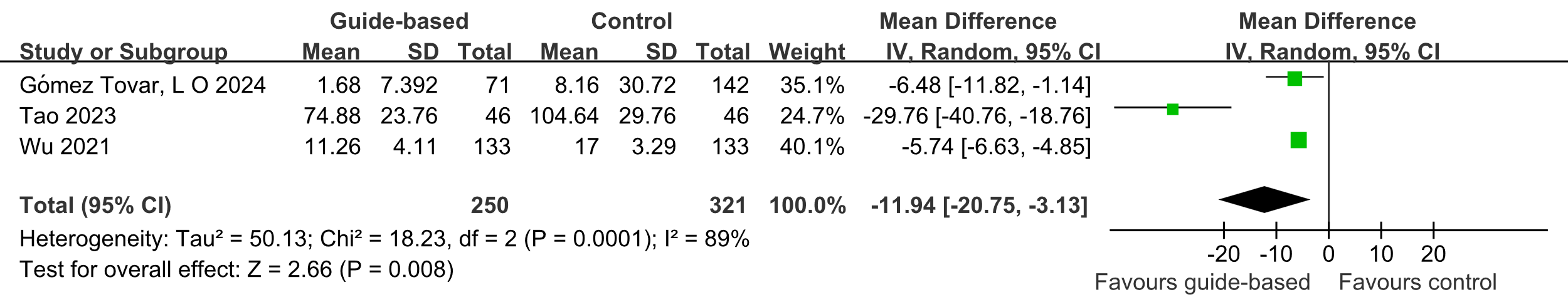


**Supplementary Figure 2: Effect of guide-based interventions on the duration of delirium with patients in ICU.**

When compared with control groups (*n* = 321), the guide-based intervention groups (*n* = 250) decreased the duration of delirium (WMD = -11.94, 95% CI -20.75 to -3.13, P = 0.008, I2=89%; random-effects model).


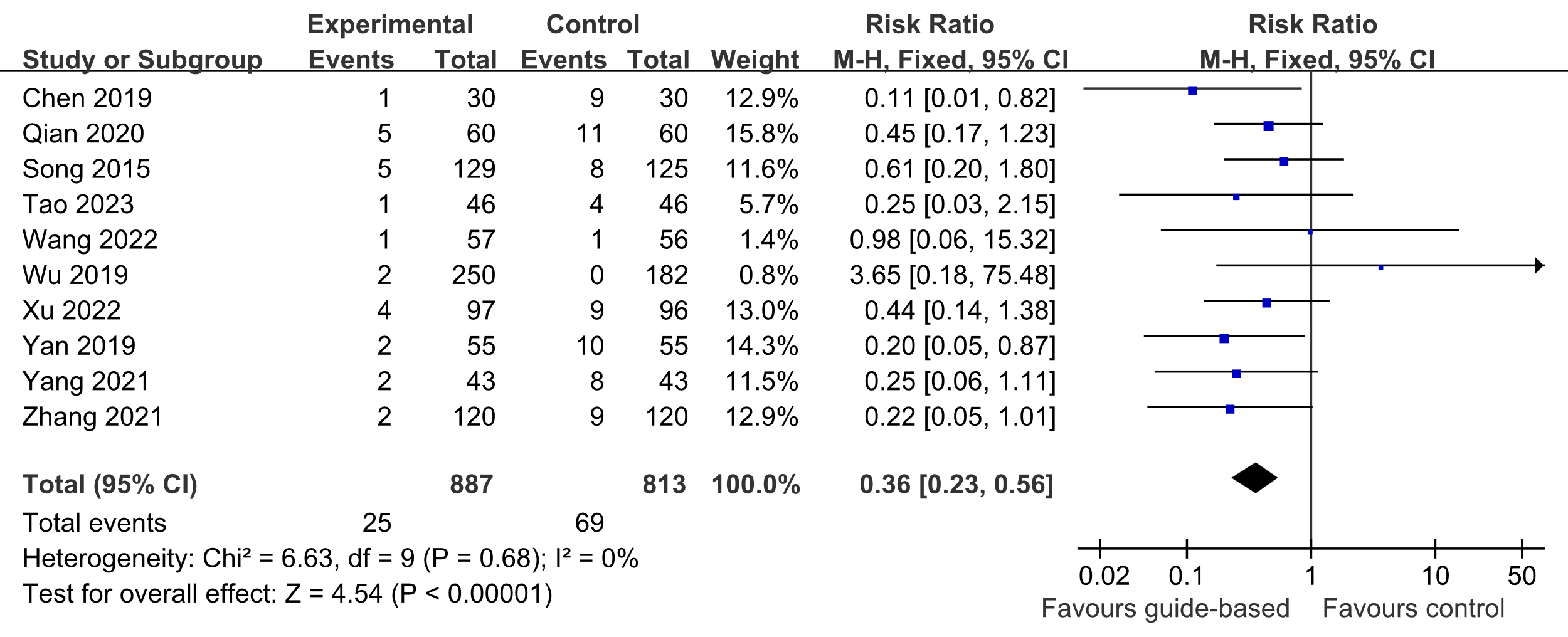


**Supplementary Figure 3: Effect of guide-based interventions on the unplanned extubation rate with patients in ICU.**

When compared with control groups (*n* = 813), the guide-based intervention groups (*n* = 887) decreased the unplanned extubation rate (RR=0.36, 95% CI 0.23 to 0.56, P＜0.001; I2=0%; ﬁxed-effects model).


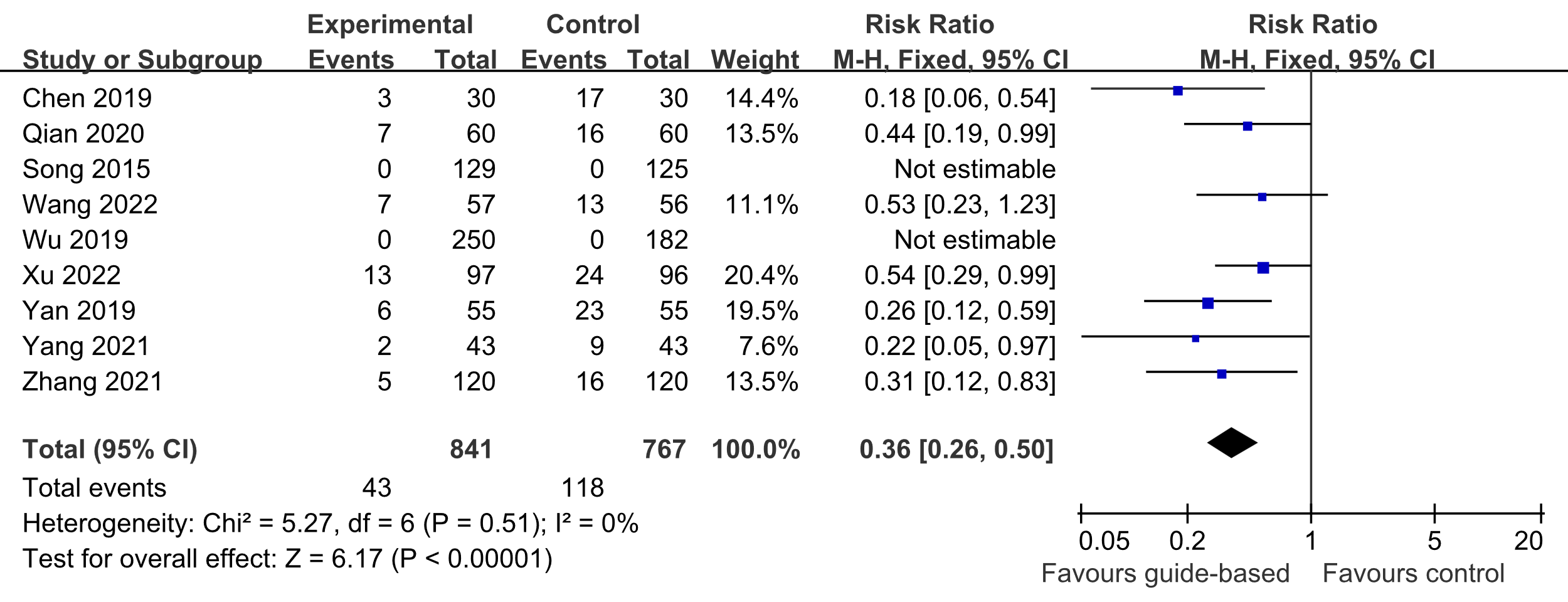


**Supplementary Figure 4: Effect of guide-based interventions on the other complications rate with patients in ICU.**

When compared with control groups (*n* = 767), the guide-based intervention groups (*n* = 841) decreased the other complications rate (RR=0.36, 95% CI 0.26 to 0.50, P＜0.001; I2=0%; ﬁxed-effects model).


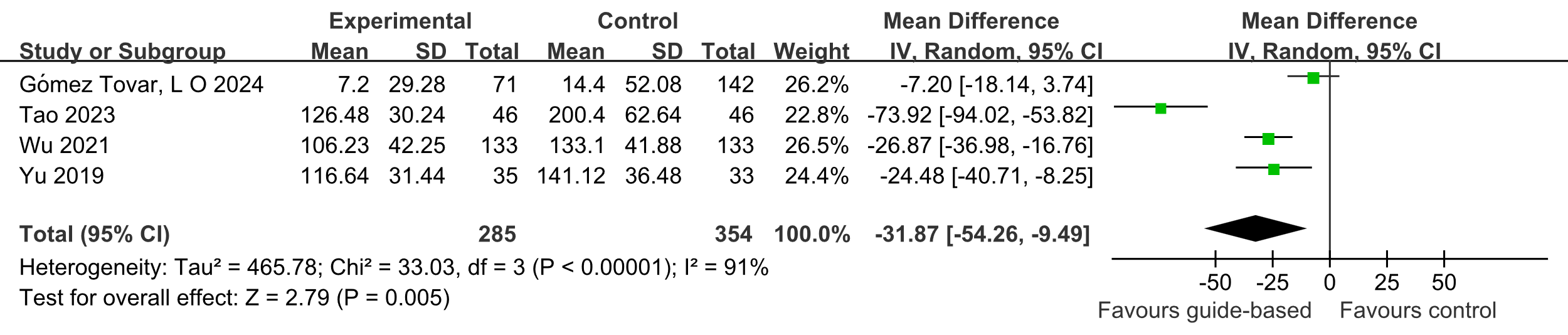


**Supplementary Figure 5: Effect of guide-based interventions on the duration of mechanical ventilation with patients in ICU.**

When compared with control groups (*n* = 354), the guide-based intervention groups (*n* = 285) decreased the duration of mechanical ventilation (WMD = -31.87, 95% CI: -54.26 to -9.49, P = 0.005, I2=91%; random-effects model).


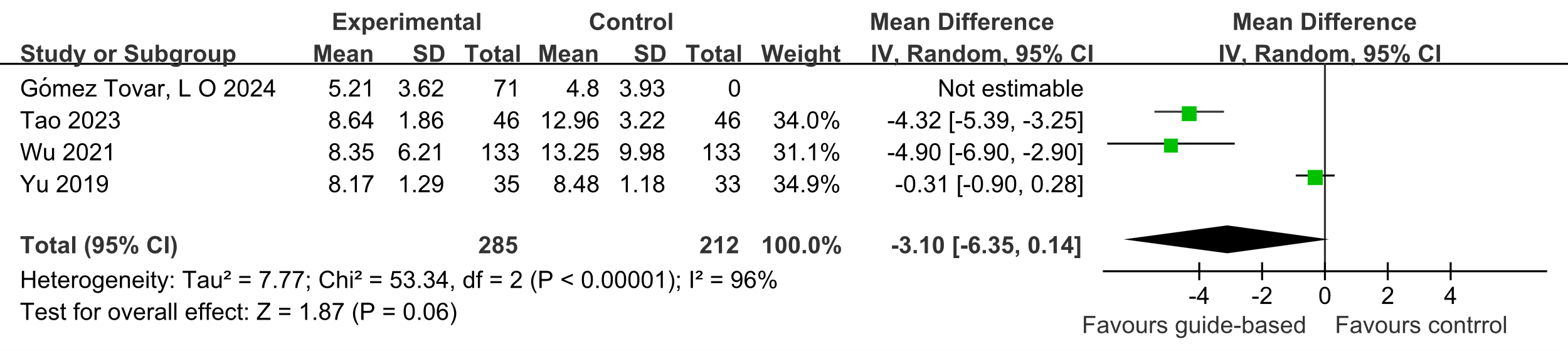


**Supplementary Figure 6: Effect of guide-based interventions on the length of stay in the ICU with patients.**

There was no difference in the length of stay in the ICU between the patients in the guide-based intervention group (n = 285) and control group (n = 212) (WMD = -31.87, 95% CI: -54.26 to -9.49, P = 0.005, I2=91%; random-effects model).


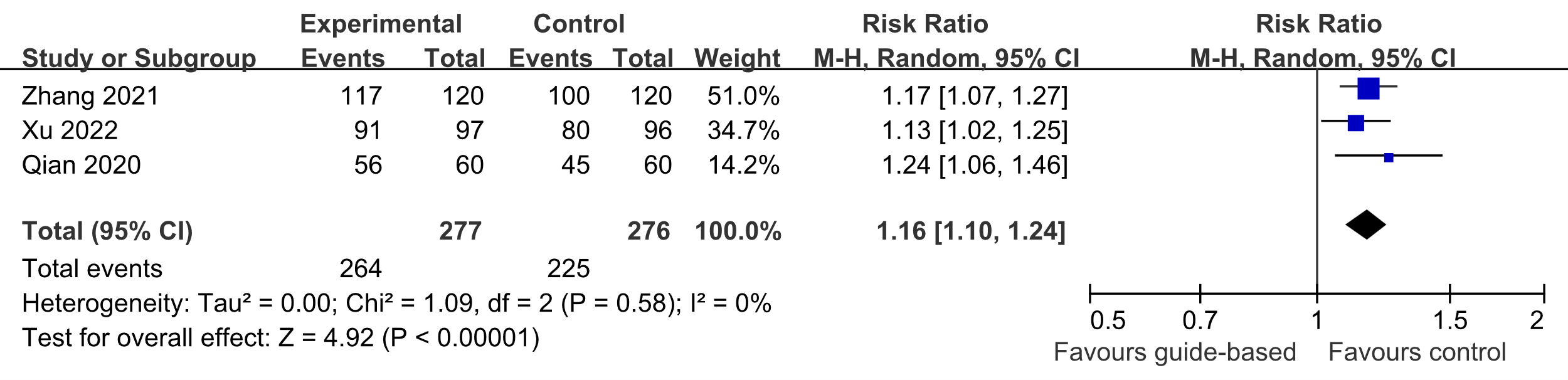


**Supplementary Figure 7: Effect of guide-based interventions on the patient satisfaction with patients in ICU.**

When compared with control groups (*n* = 276), the guide-based intervention groups (*n* = 277) increased the patient satisfaction (RR=1.16, 95% CI 1.10 to 1.24, P＜0.001; I2=0%; random-effects model).


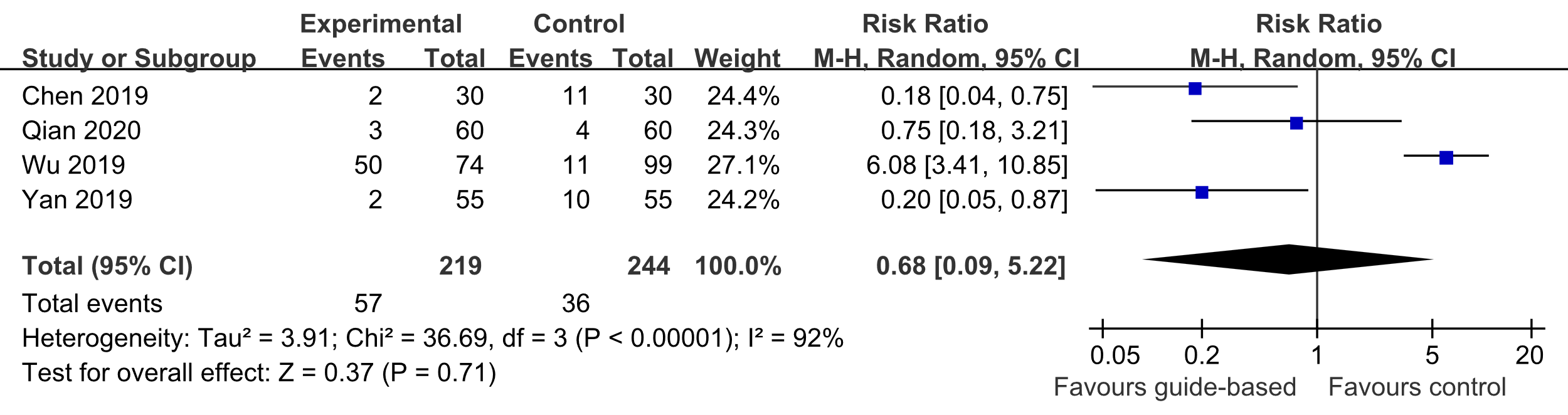


**Supplementary Figure 8: Effect of guide-based interventions on the patient agitated or anxiety rate with patients in ICU.**

There was no difference in patient agitated or anxiety rate between the patients in the guide-based intervention group (n = 244) and control group (n = 219) (RR=0.68, 95% CI 0.09 to 5.22, P=0.71; I2=92%; random-effects model).


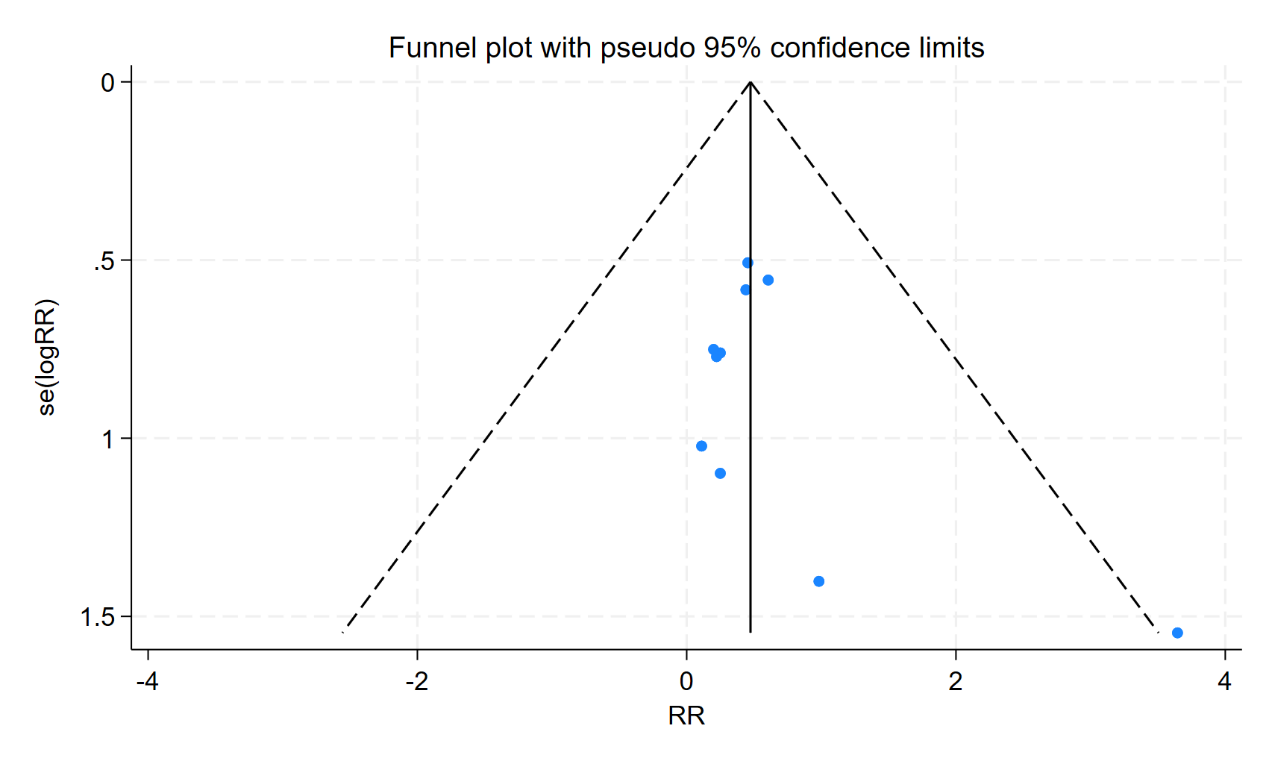
 **Supplementary Figure 9. The funnel plot of** **unplanned extubation rate**

(Egger test, P = 0.199; Begg test, P = 0.721)


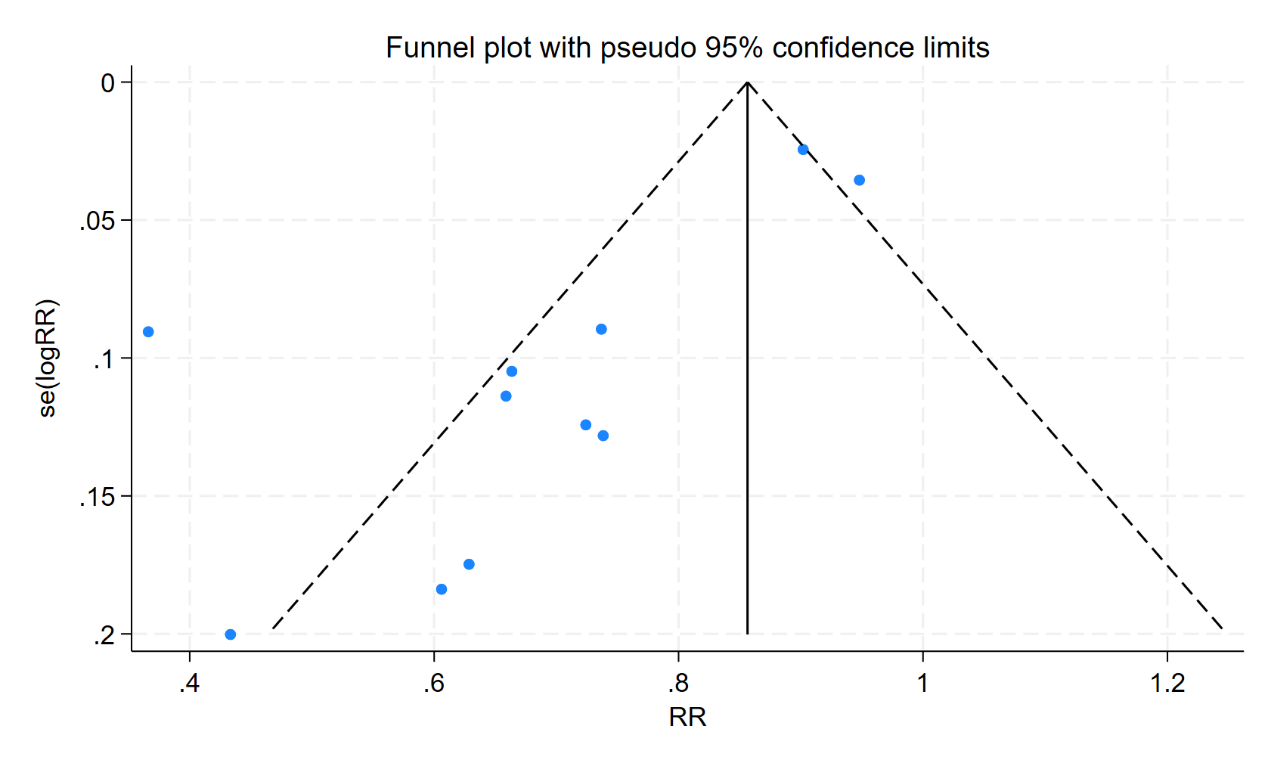
 **Supplementary Figure 10. The funnel plot of PR rate**

(Egger test, P = 0.004; Begg test, P = 0.35)


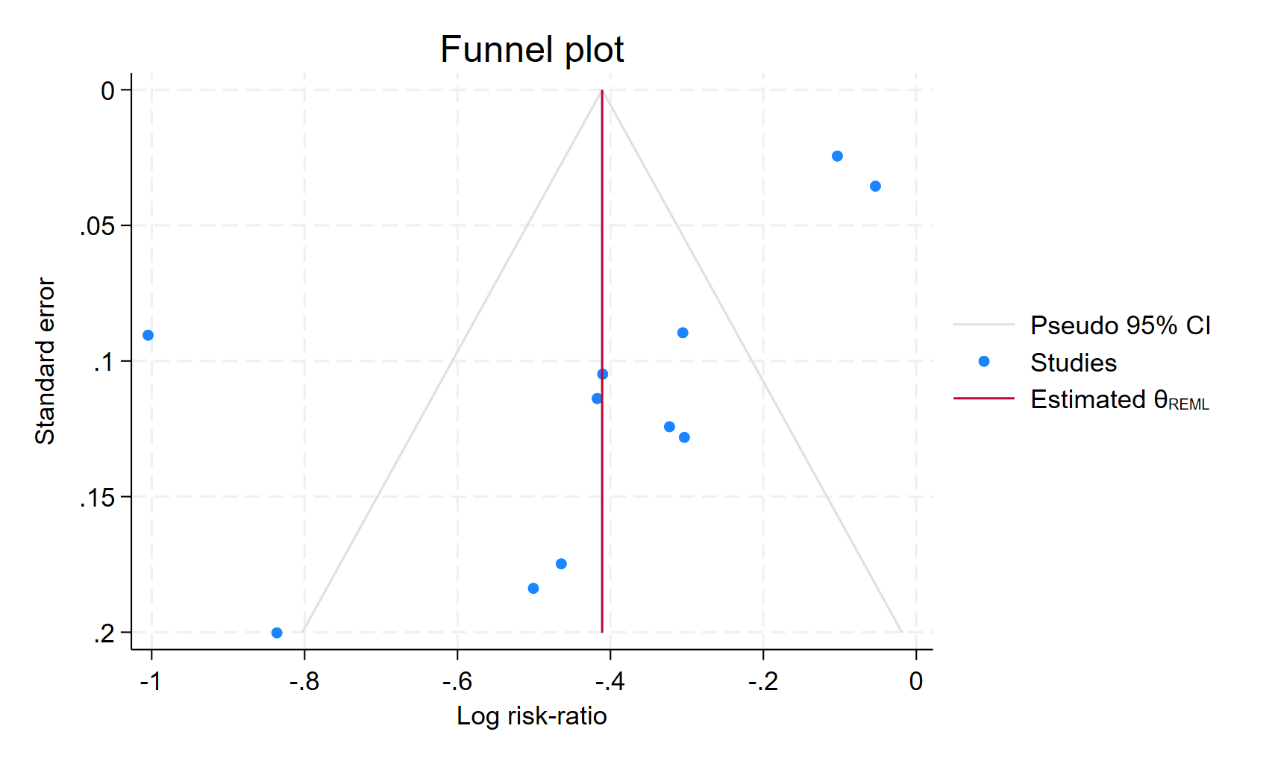


**Supplementary Figure 11. The funnel plot of the incidence of PR rate**

**Supplementary Table 1. Effects of the incidence of PR rate by trim-and fill method**

| **Studies** | **RR** | **95%Cl** |
| --- | --- | --- |
| **Observed** | **-0.411** | **-0.585, -0.236** |
| **Observed + Imputed** | **-0.411** | **-0.585, -0.236** |
